# Entangling capacities and the geometry of quantum operations

**DOI:** 10.1038/s41598-020-72881-z

**Published:** 2020-09-29

**Authors:** Jhih-Yuan Kao, Chung-Hsien Chou

**Affiliations:** 1 Department of Physics, National Cheng Kung University, Tainan, 70101 Taiwan; 2 Center for Quantum Frontiers of Research and Technology, NCKU, Tainan, 70101 Taiwan

**Keywords:** Quantum information, Quantum mechanics

## Abstract

Quantum operations are the fundamental transformations on quantum states. In this work, we study the relation between entangling capacities of operations, geometry of operations, and positive partial transpose (PPT) states, which are an important class of states in quantum information. We show a method to calculate bounds for entangling capacity, the amount of entanglement that can be produced by a quantum operation, in terms of negativity, a measure of entanglement. The bounds of entangling capacity are found to be associated with how non-PPT (PPT preserving) an operation is. A length that quantifies both entangling capacity/entanglement and PPT-ness of an operation or state can be defined, establishing a geometry characterized by PPT-ness. The distance derived from the length bounds the relative entangling capability, endowing the geometry with more physical significance. We also demonstrate the equivalence of PPT-ness and separability for unitary operations.

## Introduction

Entanglement has been found to be a useful resource for various tasks in quantum information^1,2^, so a problem arises: How to create entanglement? As an aspect of quantum states, this is the same as discerning what quantum processes/channels/operations^3–7^ can effectively produce this valuable resource, because operations govern how a state evolves or changes. There have been many studies on this problem, from various perspectives, such as how much entanglement an operation is able to produce/erase at most, on average, or per unit time^8–18^ and what operations can produce the most entanglement (perfect entangler)^19–22^. Unitary operations are usually considered^8–10,13–17,21,22^, while sometimes general quantum or Gaussian operations are investigated^11,18^, with respect to various measures.

As the more general mappings dictating quantum processes, quantum operations are completely positive (CP), and they can be deterministic (such as conventional unitary evolutions) or probabilistic (such as measurements), but either way are trace-preserving (TP) as a whole^23,24^ in order that a density operator remains a density operator after quantum operations. A probabilistic operation *S* is composed of multiple CP maps $S_i$, and the probability that a sub-operation $S_i$ is applied is $p_i=\mathrm{tr}S_i(\rho )$^24^.

PPT states and operations have a profound importance in entanglement theory; for example, it was found no entanglement can be distilled from PPT states^25^. This class of states/operations is the subject of numerous studies, e.g. how to utilize them, and whether they are Bell nonlocal^25–33^.

In this work, we quantify the capability of a quantum operation to produce entanglement by *entangling capacity*, defined as the maximal entanglement measure that can be created by a quantum operation^10,13,15,16,18^. It was found the existence of an ancilla, a system on which the operation isn’t directly applied, may help boost entangling capacity^10,13,18^. One can thus define the entangling capacity of an operation *S* assisted by an ancilla with respect to an entanglement measure *m*:

1 $$\begin{aligned} \text {EC}_m(S):=\max _\rho \{m({\mathscr {I}}\otimes S(\rho ))-m(\rho )\}, \end{aligned}$$

where ${\mathscr {I}}$ is the identity mapping on the ancilla. For a probabilistic operation *S*, define the (average) entangling capacity assisted by an ancilla as

2 $$\begin{aligned} \text {EC}_m(S):=\max _\rho \left\{ \sum _i p_i m({\mathscr {I}} \otimes S_i(\rho ))-m(\rho ) \right\} . \end{aligned}$$

Note the maximizations in (2) are over all density operators $\rho$ of the composite system, comprising the original system and the ancilla.

We will obtain bounds (Proposition 1) for entangling capacities in terms of negativities. Since negativities bound teleportation capacity and distillable entanglement^1,25,34–36^, our results give bounds for teleportation capacity and distillable entanglement that can be created by quantum operations.

Qualitatively, it is known that a PPT operation can’t create negativity out of a PPT state^3,25^, and in this work, we would like to investigate the quantitative importance of PPT-ness of operations—A length, or norm associated with the bounds and PPT-ness, can be defined, by which, along with the distance or metric induced from it, we can provide entangling capacities of operations a geometric meaning. A strongly non-PPT operation, i.e. an operation that is “longer” in this norm, has the potential to create more negativity. In addition, the distance between operations can bound their relative entangling capability (Proposition 2). Therefore, this geometry of operations has physical importance.

A method to find bounds of entangling capacity in terms of negativities was proposed in Ref.^18^. We will compare his approach with ours, and show that, albeit quite dissimilar in form, our bounds can lead to his. Whenever there are bounds, it is natural to ask whether or when they can be saturated. Proposition 4 will answer this question, and we will lay out a procedure to find the states with which to reach the bound. In addition, we are able to show PPT-ness and separability are equivalent for unitary operations, similar to pure states (Proposition 3).

The result of this work can be applied to systems of any finite dimensions, so it may be useful in the study of quantum processes that utilizes high-dimensional spaces^37^. A list of symbols and acronyms is compiled; see Table 1. The reader may refer to the supplemental material S1 accompanying this paper or J.-Y. Kao’s PhD thesis^38^ for details, derivations and a more rigorous approach to this study.

**Table 1:** List of symbols and acronyms.

CP	Completely positive
TP	Trace-preserving
HP	Hermiticity-preserving
PPT	Positive partial transpose (preserving)
$\text {EC}_m$	Entangling capacity w.r.t. measure *m*
*L*	A linear mapping from operators to operators
*O*	A generic operator
${\mathscr {I}}$	Identity mapping on operators
*I*	Identity operator
$\widetilde{L}_{\pm }$	*L* as the difference of two CP mappings
$L_{\pm }$	Like above, but by eigendecomposing (4)
*S* and $S_i$	Quantum operations and sub-operations
*T*, $O^T$	Transposition and transpose
$\Gamma$, $O^\Gamma$, $L^\Gamma$	Partial transposition and partial transposes; $L^\Gamma :=\Gamma \circ L\circ \Gamma$
$S^\Gamma _{\pm }$	${S^\Gamma }_{\pm }$
${S_i^\Gamma }_{\pm }^\dagger$	${{{S_i}^\Gamma }_{\pm }}^\dagger$
$\widetilde{S_i^\Gamma }_{\pm }$ and ${S_i^\Gamma }_{\pm }$	Like $\widetilde{L}_{\pm }$ and $L_{\pm }$
$d_i$	Dimension of system *i*
$\ker$	The kernel or null space
ran	The range/image of a mapping

## Preliminaries

### Linear mappings

In this work, a “linear mapping” always refers to one that maps an operator to another operator. A generic linear mapping will be denoted by *L*. Quantum operations are CPTP linear mappings, which will be denoted by *S*.

#### Choi isomorphism

For a linear mapping *L*, Choi^39–42^ showed that with a mapping ${\mathscr {T}}$ defined as

3 $$\begin{aligned} {\mathscr {T}}(L):=\sum _{i,j} |{a_i}\rangle \langle {a_j}| \otimes L(|{a_i}\rangle \langle {a_j}|), \end{aligned}$$

where $\{|{a_i}\rangle \}$ is an orthonormal basis, *L* is CP if and only if ${\mathscr {T}}(L)\ge 0$^41^. Conversely, *L* can be expressed in terms of ${\mathscr {T}}(L)$:^42^

4 $$\begin{aligned} L(O)=\mathrm{tr}_1 (O^{\mathrm{T}}\otimes I {\mathscr {T}}(L)), \end{aligned}$$

where the transposition is on the basis $\{|{a_i}\rangle \}$, and the partial trace $\mathrm{tr}_1$ is with respect to the first party, i.e. the party to the left of $\otimes$ in Eq. (3).

When there are two parties A and B involved, (3) becomes

5 $$\begin{aligned} {\mathscr {T}}(L)=\sum _{i,j,k,l} |{a_i}\rangle \langle {a_j}|\otimes |{b_k}\rangle \langle {b_l}|\otimes L(|{a_i}\rangle \langle {a_j}|\otimes |{b_k}\rangle \langle {b_l}|), \end{aligned}$$

where $\{|{a_i}\rangle \}$ and $\{|{b_j}\rangle \}$ are orthonormal bases of A and B.

#### Operator-sum representation

To obtain an operator-sum representation^5,23,41^ of a linear mapping *L*, suppose the eigenvalues of ${\mathscr {T}}(L)$ are $c_i$ with corresponding eigenvectors $|{v_i}\rangle =\sum _{jk} d_{jk}^i |{a_j}\rangle |{e_k}\rangle ,$ where $\{e_i\}$ is an orthonormal basis. Define

6 $$\begin{aligned} V_i:= \sum _{j,k} d^i_{jk}|{e_k}\rangle \langle {a_j}|, \end{aligned}$$

and it turns out that

7 $$\begin{aligned} L(O)=\sum _i c_i V_i O V_i^\dagger \end{aligned}$$

is an operator-sum representation of *L*.

#### Hilbert–Schmidt inner product

We can express the trace of an operator *O* after a linear mapping *L* as a Hilbert–Schmidt inner product^39,40^:

8 $$\begin{aligned} \mathrm{tr}L(O)=(I|L(O))=(L^\dagger (I)|O). \end{aligned}$$

*L* is TP if and only if $L^\dagger (I)=I$. $L^\dagger (I)$ can be obtained via the operator-sum representation (7): $L^\dagger (I)=\sum _i c_i V_i^\dagger V_i$, or by Lemma 3 from “Methods”.

#### Hermiticity-preserving mappings

Any Hermiticity-preserving (HP) mapping *L* can be decomposed as^39,41^

9 $$\begin{aligned} L=\widetilde{L}_+-\widetilde{L}_-,\text { where }\widetilde{L}_{\pm }\text { are CP.} \end{aligned}$$

Given any operator *O*, we can find the “positive” and “negative” parts of *O* by spectral decomposition:

10 $$\begin{aligned} O=O^+-O^-,\,O^\pm \ge 0. \end{aligned}$$

We can similarly eigendecomposing ${\mathscr {T}}(L)$ of Eq. (3): ${\mathscr {T}}(L)=({\mathscr {T}}(L))^+-({\mathscr {T}}(L))^-$^43^, and Eq. (4) shows how they act on any operator *O*:

11 $$\begin{aligned} L_{\pm } (O)= \mathrm{tr}_1\left( O^{\mathrm{T}}\otimes I ({\mathscr {T}}(L))^\pm \right) . \end{aligned}$$

As $({\mathscr {T}}(L))^\pm \ge 0$, $L_{\pm }$ are also CP, so $L_{\pm }$ obtained this way are a special case of Eq. (9).

### Norms of operators, partial transposition and negativities

The trace norm is defined as^35,44–47^

12 $$\begin{aligned} ||O||_1:=\mathrm{tr}|O|=\mathrm{tr}\sqrt{O^\dagger O}. \end{aligned}$$

For a Hermitian operator *H*, its operator norm ||*H*||^45–50^ is

13 $$\begin{aligned} ||H||=\max (|h_i|:h_i's\text { are eigenvalues of }H). \end{aligned}$$

Transposition *T* is a positive but non-CP mapping^43,51^; namely partial transposition

14 $$\begin{aligned} \Gamma :=T\otimes {\mathscr {I}} \end{aligned}$$

is a non-positive linear mapping. The partial transpose of an operator *O* is often denoted by

15 $$\begin{aligned} O^\Gamma :=\Gamma (O)=(T\otimes {\mathscr {I}})O. \end{aligned}$$

Both *T* and $\Gamma$ are HP and TP^5^. A state $\rho$ is said to be a PPT (positive partial transpose) state if $\rho ^\Gamma \ge 0$. A separable state must be PPT by Peres–Horodecki criterion^26,51^. Negativity and logarithmic negativity^35^ are both entanglement measures (monotones) and can be considered as quantitative versions of Peres criterion^5,35,52,53^, defined respectively as

16 $$\begin{aligned} E_N&:=||{\rho ^\Gamma }^-||_1=(||\rho ^\Gamma ||_1-1)/2,\nonumber \\ E_L&:=\log ||\rho ^\Gamma ||_1. \end{aligned}$$

The partial transpose of a linear mapping *L* is defined as^24,30,54^

17 $$\begin{aligned} L^\Gamma (O):= L(O^\Gamma )^\Gamma , \text { or } L^\Gamma := \Gamma \circ L\circ \Gamma . \end{aligned}$$

Transposition is linear, so is $L^\Gamma .$ An operation *S* for which $S^\Gamma$ is CP, i.e. ${\mathscr {T}}(S^\Gamma )\ge 0$, is called a PPT (PPT preserving) operation, as it maps a PPT state to another one^3,24,30,54,55^.

## Results and discussions

### Bounds for entangling capacities

#### Proposition 1

*(Note the upper bounds for* $\mathrm {EC}_N(S)$*below are actually maximized over all states* $\rho$*with a given* $||\rho ^\Gamma ||_1$, *in contrast with the definition* (1) *and* (2))

1. *There exist upper and lower bounds for entangling capacities of deterministic operations:*
  18 $$\begin{aligned} \frac{||{S^\Gamma _-}^\dagger (I)||_1}{d_A d_B}&\le \mathrm {EC}_N(S) \le ||\widetilde{S^\Gamma }_-^\dagger (I)||\,||\rho ^\Gamma ||_1,\nonumber \\ \log \frac{||{\mathscr {T}}(S^\Gamma )||_1}{d_A d_B}&=\log \left( 1+2\frac{||{S^\Gamma _-}^\dagger (I)||_1}{d_A d_B}\right) \le \mathrm {EC}_L(S)\le \log (1+2||\widetilde{S^\Gamma }_-^\dagger (I)||). \end{aligned}$$
2. *For a probabilistic operation composed of sub-operations* $S_i$,
  19 $$\begin{aligned} \sum _i\frac{||{S_i^\Gamma }_-^\dagger (I)||_1}{d_A d_B}&\le \mathrm {EC}_N(S)\le ||\sum _i\widetilde{S^\Gamma _i}^\dagger _-(I) ||\,||\rho ^\Gamma ||_1,\nonumber \\ \sum _{i}\frac{\mathrm{tr}{\mathscr {T}}(S_i)}{d_A d_B} \log \frac{||{\mathscr {T}}(S^\Gamma _i)||_1}{\mathrm{tr}{\mathscr {T}}(S_i)}&\le \mathrm {EC}_L(S) \le \log (1+2||\sum _i\widetilde{S^\Gamma _i}^\dagger _-(I)||). \end{aligned}$$
  *The upper bounds of part 2 can be applied to a deterministic operation* $S=\sum _i S_i$.
3. *With an initial negativity* $E_N$, *the expected negativity, i.e. probability times* $p_i$*the actual negativity* ${E_N}_i$, *after a sub-operation* $S_i$*is bounded by:*
  20 $$\begin{aligned} p_i {E_N}_i\le E_N\left( ||\widetilde{S^\Gamma _i}_-^\dagger (I)|| +||\widetilde{S^\Gamma _i}_+^\dagger (I)||\right) +||\widetilde{S^\Gamma _i}_-^\dagger (I)||. \end{aligned}$$
  *The entangling capacity of a sub-operation is bounded from below by:*
  21 $$\begin{aligned} \mathrm {EC}_N(S_i)&\ge \frac{\mathrm{tr}{S_i^\Gamma }_-^\dagger (I)}{\mathrm{tr}S_i^\dagger (I)}=\left( \frac{\mathrm{tr}{S_i^\Gamma }_+^\dagger (I)}{{\mathrm{tr}S_i^\Gamma }_-^\dagger (I)}-1\right) ^{-1},\nonumber \\ \mathrm {EC}_L(S_i)&\ge \log \left( 1+2\frac{\mathrm{tr}{S^\Gamma _i}_-^\dagger (I)}{\mathrm{tr}S_i^\dagger (I)}\right) =\log \left[ 1+2\left( \frac{\mathrm{tr}{S_i^\Gamma }_+^\dagger (I)}{\mathrm{tr}{S_i^\Gamma }_-^\dagger (I)} -1\right) ^{-1}\right] . \end{aligned}$$
  *All the upper bounds remain the same after the addition of an ancilla:* $||({\mathscr {I}}\otimes \widetilde{S_i^\Gamma }_-)^\dagger (I)||=||\widetilde{S_i^\Gamma }^\dagger _-(I)||$.

How entangling an operation can be in terms of negativities is associated with the norms of ${S^\Gamma _i}_-^\dagger (I)$. They vanish if and only if the operations are PPT, as an PPT operation on average can not increase the negativities of any state^35,53^. With a small or zero entangling capacity with respect to negativities, even if the operation is entangling, it produces mostly or entirely bound entanglement^3,25^.

For a deterministic operation $S=\sum _i S_i$, the upper bounds from (19) can be regarded as a special case of (18), by choosing $\widetilde{S^\Gamma }_{\pm }=\sum _i \widetilde{S_i^\Gamma }_{\pm }.$ With $S=p S_1+(1-p)S_2$, where both $S_i$ are TP and $0\le p\le 1$, if $S_2$ is PPT, Proposition 1 suggests that *S* is at most about *p* times as entangling as $S_1$ is—Mixing an operation with a PPT one in general makes it less entangling.

From part 3 of Proposition 1, if a sub-operation is PPT, e.g. LOCC^54^, whether its negativity can increase depends on $||{S_i^\Gamma }^\dagger (I)||/p_i.$ If the state is initially PPT, $S_i$ should be non-PPT for any negativity to be produced, and the amount is bounded by $||{S^\Gamma _i}^\dagger _-(I)||/p_i$; in other words, no entanglement can be distilled out of a PPT state after PPT (sub-)operations^3,25^.

### A geometrical point of view

#### A norm that quantifies entangling capacity and entanglement

Let us focus on deterministic operations. With a one-to-one homomorphism between two vector spaces $l:V\rightarrow W$ and a norm *p* for *W*, $p\circ l$ is a norm for *V*^49^. Thus we can define such norms for any operator or linear mapping *X*:

22 $$\begin{aligned} ||X||_{n,\Gamma }:=||X^\Gamma ||_n, \end{aligned}$$

where

23 $$\begin{aligned} ||L||_n:=||{\mathscr {T}}(L)||_n \end{aligned}$$

for any linear mapping *L*, because partial transposition is one-to-one.

$||\rho ||_{1,\Gamma }$ decides the negativities of a state, and $||S||_{1,\Gamma }$ the lower bounds for entangling capacities by Eq. (18), which can be taken for the negativity or entanglement of the operation (if normalized), because $||{\mathscr {T}}(S^\Gamma )||_1=||({\mathscr {T}}(S))^\Gamma ||_1$^43,56^. In addition, the upper bound of entangling capacity from Proposition 1 is bounded by $||S||_{1,\Gamma }$:

24 $$\begin{aligned} ||{S^\Gamma _-}^\dagger (I)||&\le ||{S^\Gamma _-}^\dagger (I)||_1\nonumber \\&=||{\mathscr {T}}(S^\Gamma _-)||_1=(||S||_{1,\Gamma }-d_A d_B)/2. \end{aligned}$$

The right hand side of the inequality is zero for PPT operations. Therefore Proposition 1 implies that the length of an operation is correlated with the extra length a state can gain after the aforementioned operation, with respect to these norms.

**Figure 1:**
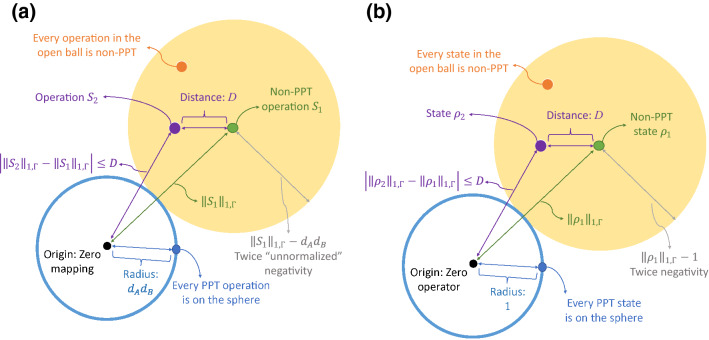
Geometry of operations and states in the spaces of linear mappings and operators, with respect to $||\cdots ||_{1,\Gamma }$. The entanglement/entangling capacity in terms of negativity is related to the length, and nearby operations/states are similarly entangling/entangled. (**a**) PPT and non-PPT operations in the space of linear mappings. (**b**) PPT and non-PPT states in the space of operators.

As a norm-induced metric,

25 $$\begin{aligned} D_{1,\Gamma }(X,Y):=||X-Y||_{1,\Gamma } \end{aligned}$$

is a distance between operations/operators *X* and *Y*. Geometrically, PPT operations are a subset of a $d_A d_B$-sphere centered at the origin with respect to $||\cdots ||_{1,\Gamma }$. By the triangle inequality, $|\; ||a||-||b|| \;|\le ||a-b||$, the length of an operation is bounded by the distance to another operation, and any operations within an open ball with center being a non-PPT operation *S* and radius $||S||_{1,\Gamma }-d_A d_B$ are non-PPT. The distance from a non-PPT operation *S* to a PPT one is at least $||S||_{1,\Gamma }-d_A d_B=2||{\mathscr {T}} (S^\Gamma )^-||_1$—It may not be the exact distance, as linear mappings $||S||_{1,\Gamma }-d_A d_B$ away from *S* are not necessarily quantum operations. All these can be applied to density operators; see Fig. 1. This is somewhat like distance-based entanglement measures^57^.

These norms are equivalent^49^. For any Hermitian operator *H* and HP mapping *L*:

26 $$\begin{aligned} \frac{||H||_1}{\min (d_A,d_B)}&\le ||H||_{1,\Gamma }\le \min (d_A,d_B)||H||_1, \end{aligned}$$

27 $$\begin{aligned} \frac{||L||_1}{\min (d_{A_1}d_{A_2},d_{B_1}d_{B_2})}&\le ||L||_{1,\Gamma } \le \min (d_{A_1}d_{A_2},d_{B_1}d_{B_2})||L||_1. \end{aligned}$$

Therefore two states or operations that are close in one norm should not be far apart in another, and vice versa. Using whichever norm does not change the topology^58^, by continuity (or linearity) of partial transposition or equivalence of norms^59,60^.

#### Significance of the distance

The distance as defined in Eq. (25) has another important physical implication beyond what’s shown in Fig. 1:

##### Proposition 2

*For any density operators* $\rho$*and* $\rho _i$, *and deterministic operations* *S* *and* $S_i$, *we have an equality*

28 $$\begin{aligned} (S_2^\Gamma -S_1^\Gamma )_+^\dagger (I)=(S_2^\Gamma -S_1^\Gamma )_-^\dagger (I), \end{aligned}$$

*and the following inequalities:*

29 $$\begin{aligned} D_{1,\Gamma }\left( S_1(\rho ),S_2(\rho )\right)&\le 2||(S_2^\Gamma -S_1^\Gamma )_{\pm }^\dagger (I)|| \, ||\rho ^\Gamma ||_1 \end{aligned}$$

30 $$\begin{aligned}&\le D_{1,\Gamma }(S_1,S_2) ||\rho ^\Gamma ||_1, \end{aligned}$$

31 $$\begin{aligned} D_{1,\Gamma }\left( S(\rho _1),S(\rho _2)\right)&\le \left( 1+2||{S^\Gamma _-}^\dagger (I)||\right) D_{1,\Gamma }(\rho _1,\rho _2). \end{aligned}$$

By the triangle inequality, this proposition provides bounds for

- the difference in negativity between different operations acting on the same state, and
- the difference in negativity between the same operation acting on different states

in terms of $D_{1,\Gamma }$. In other words,

- if two operations are close relative to $D_{1,\Gamma }$, they have similar capabilities in changing the negativity or entanglement of any state;
- if two states are close relative to $D_{1,\Gamma }$, then their negativites won’t differ much after they’re acted upon by the same operation.

**Figure 2:**
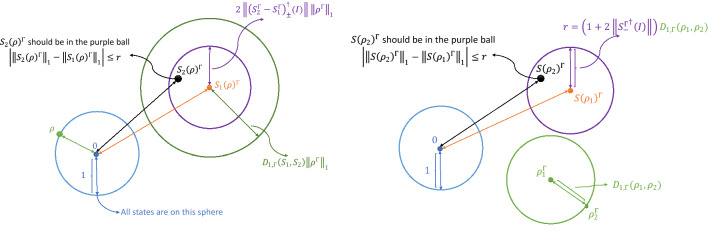
Proposition 2 and its implication in the space of operators with respect to trace norm $||\cdots ||_1$. Similar diagrams can be drawn in terms of $||\cdots ||_{1,\Gamma }$.

See Fig. 2 for a geometric presentation of Proposition 2. In short, the norm $||\cdots ||_{1,\Gamma }$ and the associated distance $D_{1,\Gamma }$ can quantify or estimate the entangling capability of quantum operations.

### Comparison between approaches

In Ref. ^18^, it was shown for a deterministic operation $S(\rho )=\sum _i V_i\rho V_i$, where $V_i$ has a Schmidt decomposition^61^ $V_i=\sum _{j(i)} \lambda _{ij} A_{ij}\otimes B_{ij}$:

32 $$\begin{aligned} \text {EC}_L(S)\le \log \left( \sum _i ||\sum _{j(i)} \lambda _{ij} A_{ij}^\dagger A_{ij}||\,||\sum _{k(i)} \lambda _{ik} B_{ik}^\dagger B_{ik}||\right) . \end{aligned}$$

It was found in Ref. ^18^ for a unitary operation with an ancilla:

33 $$\begin{aligned} \text {EC}_L(S)\ge \log \frac{(\sum _i\lambda _i)^2}{d_A d_B}, \end{aligned}$$

By Lemma 5 from the “Methods”, the upper bound can be derived from Proposition 1 and the lower bound is the same as ours.

### PPT-ness and separability of unitary operations

By Peres–Horodecki criterion, separable states are a subset of PPT states^26,51^; similarly, separable operations^24,62^ are a subset of PPT operations^54^. Additionally, all PPT pure states are separable, so only for mixed states are PPT-ness and separability distinct properties^35,63,64^. We find an analogy for unitary operations:

#### Proposition 3

*In a finite-dimensional system, the following statements are equivalent for any unitary operation* $S(O)=UOU^\dagger$, *where* *U* *is a unitary operator:*

1. *The (Schmidt) rank of* *U* *is 1*.
2. *S* *is separable*.
3. *S* *is PPT*.

Hence, any non-separable unitary operation, being PPT as well, can create negativity according to Proposition 1. In other words, only for mixed states^65^ and non-unitary operations^54,65^ are PPT-ness and separability two different properties.

### Basic unitary operators and exact entangling capacity

Basic unitary operators are those whose Schmidt decompositions $U=\sum _i \lambda _{i} A_{i}\otimes B_{i}$ have all the $A_i$ and $B_i$ proportional to unitary operators^18^. It was shown that all $2\otimes 2$ unitary operators are basic^18^, and that for a basic unitary operator, the upper and lower bounds for the entangling capacity are identical (with $\widetilde{S^\Gamma }_{\pm }=S^\Gamma _{\pm }$), so they are the exact entangling capacity^18^, which by Proposition 1 implies $\mathrm{tr}{S^\Gamma _-}^\dagger (I)=d_A d_B||{S^\Gamma _-}^\dagger (I)||,$ so

34 $$\begin{aligned} {S^\Gamma _{\pm }}^\dagger (I)\propto I. \end{aligned}$$

More generally, for deterministic operations:

#### Proposition 4

*Suppose* $\widetilde{S^\Gamma }_{\pm }=S^\Gamma _{\pm }.$*Let* $\mathrm {ran}$*denote the range or image of a mapping, and*

35 $$\begin{aligned} S^\Gamma _{\pm }(O)=\sum _i c_i^\pm V_i^\pm O {V_i^\pm }^\dagger ,\,c_i^\pm >0, \end{aligned}$$

*be operator-sum representations of* $S^\Gamma _{\pm }$, *and*

36 $$\begin{aligned} {\rho ^\Gamma }^\pm =\sum _{i} |{\psi ^\pm _i}\rangle \langle {\psi ^\pm _i}| \end{aligned}$$

*be ensembles of* ${\rho ^\Gamma }^\pm$. *The upper bound of the entangling capacity given by Proposition* 1*is reached if and only if the two conditions below are both satisfied:*

1. $\mathrm {ran}\left[ S^\Gamma _-({\rho ^\Gamma }^+) +S^\Gamma _+({\rho ^\Gamma }^-)\right]$*and* $\mathrm {ran} \left[ S^\Gamma _+({\rho ^\Gamma }^+)+S^\Gamma _-({\rho ^\Gamma }^-)\right]$*are orthogonal, which is equivalent to the orthogonality of the following vectors:*
  37 $$\begin{aligned} \langle {V_i^\pm \psi _j^\mp }|{V_k^+ \psi _l^+}\rangle =0 \text { and } \langle {V_i^\pm \psi _j^\mp }|{V_k^- \psi _l^-}\rangle =0 \;\forall i,j,k,l. \end{aligned}$$
2. $\mathrm {ran}\rho ^\Gamma$*is a subspace of the eigenspace relative to the largest eigenvalue of* ${S^\Gamma _{\pm }}^\dagger (I)$.

*The second condition above is satisfied by any state* $\rho$*when* ${S^\Gamma _{\pm }}^\dagger (I)\propto I$. *Besides, the upper and lower bounds in* (18) *are the same if and only if* ${S^\Gamma _{\pm }}^\dagger (I)\propto I$.

If ${S^\Gamma _{\pm }}^\dagger (I)\propto I$, it does not matter whether *S* is unitary or even basic—the exact entangling capacity is acquired, and $||S||_{1,\Gamma }$ reflects its true entangling capacity. The other way around, we can show a basic unitary operation always satisfies ${S^\Gamma _{\pm }}^\dagger (I)\propto I$, so the upper and lower bounds are exact.

#### Application to pure separable states

If the state $\rho$ is PPT, there will be no $|{\tilde{\psi }_i^-}\rangle$ in Eq. (37). Let’s further assume the state is pure and separable: $\rho =|{\psi }\rangle \langle {\psi }|$ and $|{\psi }\rangle =|{\psi _1}\rangle |{\psi _2}\rangle$. Given an orthonormal basis $\{|{a_i}\rangle \}$ of A, the partial transpose of $|{\psi }\rangle$ is still pure and separable:

38 $$\begin{aligned} (|{\psi }\rangle \langle {\psi }|)^\Gamma =|{\psi _1^*}\rangle \langle {\psi _1^*}|\otimes |{\psi _2}\rangle \langle {\psi _2}|, \end{aligned}$$

where $|{\psi _1}\rangle =\sum c_i|{a_i}\rangle$ and $|{\psi _1^*}\rangle =\sum c_i^*|{a_i}\rangle .$ Hence, ${\rho ^\Gamma }^+=|{\psi _1^*}\rangle \langle {\psi _1^*}|\otimes |{\psi _2}\rangle \langle {\psi _2}|$ and ${\rho ^\Gamma }^-=0,$ and (37) becomes

39 $$\begin{aligned} (\langle {\psi _1^*}|\langle {\psi _2}|){V_i^-}^\dagger V_j^+ (|{\psi _1^*}\rangle |{\psi _2}\rangle )=0\;\forall i,j. \end{aligned}$$

Consider a $2 \otimes 2$ unitary operator:

40 $$\begin{aligned} U=\left( \begin{array}{cccc} \cos \alpha &{} \sin \alpha &{}0 &{}0\\ -\sin \alpha &{} \cos \alpha &{}0 &{}0\\ 0&{}0&{}\cos \beta &{}\sin \beta \\ 0&{}0&{}-\sin \beta &{}\cos \beta \end{array}\right) . \end{aligned}$$

Being basic, we can find:

41 $$\begin{aligned} {S^\Gamma _-}^\dagger (I)=\frac{\left| \sin (\beta -\alpha )\right| }{2} I\propto I. \end{aligned}$$

With $|{\psi _i}\rangle =\cos \theta _i|{\uparrow }\rangle +e^{i\phi _i} \sin \theta _i|{\downarrow }\rangle$ the solution to Eq. (39) is $\theta _1=\pi /4+n\pi /2$, and $\theta _2=m\pi /2$ or $\phi _2=p\pi$, with $m,n,p\in {\mathbb {Z}}$. Any pure separable states that satisfy this condition, e.g. $(1,0,1,0)/\sqrt{2}$ will have

42 $$\begin{aligned} E_N=\frac{\left| \sin (\beta -\alpha )\right| }{2} \text { and }E_L =\log (1+\left| \sin (\beta -\alpha )\right| ) \end{aligned}$$

after this unitary operation according to Propositions 1 and 4, and Eq. (42) is also the maximal negativities any state can gain under *U*.

Now consider a $2\otimes 3$ unitary operator:

43 $$\begin{aligned} \left( \begin{array}{c|c} I_{3}&{}0_{3}\\ \hline 0_{3}&{}\begin{array}{ccc} \cos \beta &{}\sin \beta &{}0\\ -\sin \beta &{}\cos \beta &{}0\\ 0&{}0&{}1 \end{array} \end{array}\right) \cdot \left( \begin{array}{c|c} I_{4}&{}0_{4}\\ \hline 0_{4}&{}\begin{array}{cc} \cos \alpha &{}\sin \alpha \\ -\sin \alpha &{}\cos \alpha \end{array} \end{array}\right) . \end{aligned}$$

In general the upper and lower bounds do not coincide; however when $\alpha =2\pi /3$ and $\beta =0$, ${S^\Gamma _-}^\dagger (I)= I/2$, so by Proposition 1 and 4 we can find the exact entangling capacities there, which are

44 $$\begin{aligned} \mathrm {EC}_N=1/2\text { and }\mathrm {EC}_L=2. \end{aligned}$$

Now let’s find pure separable states that reach the entangling capacities, i.e. to solve Eq. (39). For simplicity assume $|{\psi _1}\rangle =\cos \theta _1|{\uparrow }\rangle +\sin \theta _1 |{\downarrow }\rangle$ and $|{\psi _2}\rangle =\cos \theta _2\sin \phi |{0}\rangle +\sin \theta _2\sin \phi |{1}\rangle +\cos \phi |{2}\rangle$, and we can find the solution will be $\sin ^2\phi \cos ^2\theta _2=1/3$ and $\cos 2\theta _1=0$, e.g. $1/\sqrt{6}(|{\uparrow }\rangle +|{\downarrow }\rangle )(|{0}\rangle +\sqrt{2}|{2}\rangle )$.

## Conclusion

In Proposition 1 we found upper and lower bounds for entangling capacities that are applicable to both deterministic and probabilistic operations, generalizing the work by Campbell^18^. Furthermore, by giving operations and states appropriate norms (lengths) and metrics (distances), Eqs. (22) and (23), we associate the bounds of entangling capacities and entanglement with geometry, as illustrated by Figs. 1 and 2. Proposition 2 provides the distance another physical importance. Note the difference between Proposition 1 and 2: Even if two operations have identical upper bounds and identical lower bounds in Eq. (18), when they act on the same state, the negativities afterwards may be wildly different if the two operations are far apart, according to Proposition 2.

Proposition 3 is interesting in that it shows PPT-ness only manifests itself as different from separability when we start to consider mixed states and non-unitary operations. Finally, Proposition 4 demonstrates the condition under which the bounds upper from Proposition 1 can be attained, as well as the condition under which the bounds become exact. We further portrayed how to find pure separable states that satisfy such conditions. Entanglement of a dynamical system can be studied with this method in a state-independent way. For example, with $\alpha (t)$ and $\beta (t)$ for the unitary operator in Eq. (40), we can investigate how entangling the system is as it evolves, and because the solution to Eq. (39) turned out to be independent of $\alpha$ and $\beta$, we know that with a proper initial state the entanglement (negativity) is maximized at any moment.

Our results also reflect that operations and states are tight-knit: The entanglement (negativity) of operations is correlated with that of states. In Ref. ^56^ a similar phenomenon was observed for entangling power in terms of linear entropy. We expect this to happen in other facets of states/operations: For example, a more non-separable operation could make states more non-separable, if a suitable measure of non-separability is used^66^.

Because PPT operations are defined to be CP after partial transposition, ancillas come in naturally: As we decompose an HP mapping into CP rather than positive parts, the upper bounds are unaltered with the addition of an ancilla. Furthermore, a non-PPT operation may be positive after partial transposition, and not be able to create any negativity due to positivity, but after adding an ancilla, it will fail to become positive after partial transposition. Studying operations through Choi isomorphism makes ancillas a natural fit.

Practically, this work could facilitate the study of quantum information in higher dimensions^37^: Proposition 1 (or the bounds shown in Ref.^18^) provides a tool for easily finding out which operations are potentially more entangling, after which we may apply Proposition 4 to discover the optimal states. As we may not be able to peform a quantum operation perfectly, Proposition 2 presents a tolerance for imperfection: For example, let’s assume the operation isn’t perfect. Suppose the perfect and imperfect (deterministic) operations are *S* and $S'$ respectively, and that the input state $\rho$ is separable. If we want the negativity of $S'(\rho )$ to be within $99.9\%$ of that of $S(\rho )$, then any operations inside the $0.1\% ||S(\rho )||_{1,\Gamma }$-closed ball centered at *S* (in terms of $||\cdots ||_{1,\Gamma }$) can be used; similarly, if we can’t prepare a perfect state, Proposition 2 can also provide the tolerance.

## Methods

Here are several lemmas relevant to the derivation.

### Lemma 1

*For any Hermitian operator* *H* *on a finite-dimensional Hilbert space* $${\mathscr {H}}$$, *among all possible such decompositions:* $$H=\widetilde{H}^+-\widetilde{H}^-,$$$$\widetilde{H}^\pm \ge 0$$, *the eigendecomposition* $$H=H^+-H^-$$**is the** **unique** *one that minimizes* $$\mathrm{tr}\widetilde{H}^+$$, $$\mathrm{tr}\widetilde{H}^-$$, *and* $$\mathrm{tr}(\widetilde{H}^++\widetilde{H}^-)$$*; minimizing any one of them is the same as minimizing each of them. A decomposition in which* $$\widetilde{{\mathscr {H}}}^\pm :=(\ker \widetilde{H}^\pm )^\perp =\mathrm {ran}\widetilde{H}^\pm $$*are orthogonal is equivalent to the eigendecomposition*.

### Lemma 2

*Suppose* $P_1$*and* $P_2$*are two positive operators, and they have such ensembles*^65,67^:

45 $$\begin{aligned} P_i=\sum _j |\psi _j^i\rangle \langle \psi _j^i |, \end{aligned}$$

*where each* $\{|\psi _j^i\rangle \}$*is a set of nonzero vectors that aren’t necessarily normalized or mutually orthogonal. Then each eigenvector/eigenspace of* $P_2$*corresponding to a nonzero eigenvalue is orthogonal to each of* $P_1$*if and only if*

$$\begin{aligned} \langle \psi _i^1|\psi _j^2\rangle =0 \;\forall i,j, \end{aligned}$$

*in other words, if and only if*

46 $$\begin{aligned} \mathrm {ran}P_1\perp \mathrm {ran}P_2. \end{aligned}$$

### Lemma 3

*For a linear mapping* *L*,

47 $$\begin{aligned} L^\dagger (I)=\mathrm{tr}_2{\mathscr {T}}(L)^*. \end{aligned}$$

*The (partial) trace is taken on the second party, i.e. to the right of* $\otimes$*in Eq.* (3).

### Lemma 4

*For an HPTP mapping* *L* *and Hermitian* *H*,

48 $$\begin{aligned} \mathrm{tr}L(H)^--\mathrm{tr}H^-&\le ||\widetilde{L}^\dagger _-(I)||\,||H||_1, \end{aligned}$$

49 $$\begin{aligned} ||L(H)||_1-||H||_1&\le 2||\widetilde{L}^\dagger _-(I)||\,||H||_1. \end{aligned}$$

### Lemma 5

*For a deterministic operation* $S=\sum _i S_i$, *where* $S_i=V_i \rho V_i^\dagger$*with the Schmidt decompositions of* $V_i$*being* $V_i=\sum _{j(i)} \lambda _{ij} A_{ij}\otimes B_{ij},$

50 $$\begin{aligned} 1+2||\sum _i {S^\Gamma _i}_-^\dagger (I)||&=||\sum _i\sum _{j(i)} \lambda _{ij}{A_{ij}^*}^\dagger A_{ij}^*\otimes \sum _{k(i)} \lambda _{ik} B_{ik}^\dagger B_{ik}|| \end{aligned}$$

51 $$\begin{aligned}&\le \sum _i ||\sum _{j(i)} \lambda _{ij} A_{ij}^\dagger A_{ij}||\,|| \sum _{k(i)} \lambda _{ik} B_{ik}^\dagger B_{ik}||. \end{aligned}$$

*For a unitary operation* $S(\rho )=U\rho U^\dagger$, *where the Schmidt decomposition of* *U* *is* $U=\sum _i \lambda _i A_i\otimes B_i$,

52 $$\begin{aligned} 1+2\frac{||{S^\Gamma _-}^\dagger (I)||_1}{d_A d_B} =\frac{(\sum _i\lambda _i)^2}{d_A d_B}. \end{aligned}$$

The upper bounds come from matrix Hölder inequality^45,68^: For $1\le p,q\le \infty$ and $1/p+1/q=1$ given two operators $O_1$ and $O_2$ we have

53 $$\begin{aligned} |\mathrm{tr}O_1^\dagger O_2|=|(O_1,O_2)|\le ||O_1||_p||O_2||_q. \end{aligned}$$

We chose $p=\infty$ and $q=1$, because negativity is determined by trace norm, and physical states have trace 1. Concavity of logarithm is also utilized^53,69^. More general bounds can be derived as well; details can be found in the supplementary material S1, which contains derivations and particulars of other essential elements for this work.

## Supplementary information

Supplementary material 1

## Data Availability

Figures required for this study can be found in the supplementary material.
