# Supplementary material for: Entangling capacities and the geometry of quantum operations
Source: Sci Rep. 2020 Sep 29;10:15978. doi: 10.1038/s41598-020-72881-z (PMC7524745; doi:10.1038/s41598-020-72881-z)
Supplement: Supplementary file 1 — Supplementary material 1 [file 41598_2020_72881_MOESM1_ESM.pdf]

Supplemental material for: Entangling Capacities and the Geometry of Quantum Operations

Jihh-Yuan Kao and Chung-Hsien Chou

Department of physics, National Cheng Kung University, Tainan 70101, Taiwan
Center for Quantum Frontiers of Research and Technology, National Cheng Kung University, Tainan 70101, Taiwan

1 Mathematical essentials and terminologies

Like in the main paper, for the reader's convenience we compile a list of symbols and abbreviations; see Table 1. If interested, a more rigorous approach to this problem can be found in J.-Y Kao's PhD thesis [1].

1.1 Linear mappings and quantum operations

We are only concerned with finite-dimensional Hilbert spaces, denoted by $\mathcal{H}$. The symbol $\mathcal{L}$ is for the vector space formed by linear mappings, e.g. $\mathcal{L}(\mathcal{H}_1, \mathcal{H}_2)$ is the space constituted by linear mappings from $\mathcal{H}_1$ to $\mathcal{H}_2$, $\mathcal{L}(\mathcal{H}) := \mathcal{L}(\mathcal{H}, \mathcal{H})$ is a space of operators, and Choi isomorphism $\mathscr{T}$ is an isomorphism between $\mathcal{L}(\mathcal{L}(\mathcal{H}_1), \mathcal{L}(\mathcal{H}_2))$ and $\mathcal{L}(\mathcal{H}_1) \otimes \mathcal{L}(\mathcal{H}_2)$ [2].

The term "linear mapping" usually refers to one from an operator algebra to another, i.e. they constitute the space $\mathcal{L}(\mathcal{L}(\mathcal{H}_1), \mathcal{L}(\mathcal{H}_2))$; the context dictates what they actually mean. The symbol L is for a linear mapping, while S and S_i are for quantum operations and sub-operations thereof: S and S_i are CP. In general we use S without a subscript for a TP (deterministic) quantum operation, but sometimes we'll be a bit loose on this, as in Sec. 5.1.

1.2 Entangling capacity

Entangling capacity of a deterministic operation or sub-operation S_i with respect to an entanglement measure m, when aided by an ancilla, is defined as [3, 4, 5, 6]

$$\mathrm{EC}_m(S_i) := \max_\rho \{m(\mathcal{I}_a \otimes S_i(\rho)) - m(\rho)\}, \tag{1}$$

where m gives the same result whether the input state is normalized or not. For a probabilistic operation S with sub-operations S_i, it's

$$\mathrm{EC}_m(S) := \max_\rho \Big\{ \sum_i p_i m(\mathcal{I}_a \otimes S_i(\rho)) - m(\rho) \Big\}. \tag{2}$$

Here the ancilla space $\mathcal{H}_a$ can be any finite-dimensional inner-product space, $\mathcal{I}_a$ is the identity mapping on $\mathcal{L}(\mathcal{H}_a)$, and the maximizations are over all density operators in $\mathcal{L}(\mathcal{H}_a \otimes \mathcal{H})$.

In Ref. [6], entangling capacity was actually defined to be the supremum, rather than maximum over all states. However, if the measure m is continuous, then since the space is finite-dimensional, physical states in the space of Hermitian operators is a compact set $\mathcal{K}$, and $m(\mathcal{K})$ is also compact [7, 8], so there's no difference between the supremum and maximum.

1.3 Hilbert-Schmidt inner product

We make use of Hilbert-Schmidt inner product: for $O_i \in \mathcal{L}(\mathcal{H}_i)$

$$(O_2|O_1) := \mathrm{tr}(O_2^\dagger O_1), \tag{3}$$

linear in the second argument, like bra-ket. It's an inner product, because it satisfies all the properties for an inner product [9]. For a linear mapping $L : \mathcal{L}(\mathcal{H}_1) \to \mathcal{L}(\mathcal{H}_2)$ [2, 10],

$$(O_2|L(O_1)) = (L^\dagger(O_2)|O_1); \tag{4}$$

CP	Completely positive
TP	Trace preserving
HP	Hermiticity preserving
PPT	Positive partial transpose (preserving)
$\mathcal{H}$	Hilbert space
EC_m	Entangling capacity with respect to measure m
$\mathcal{L}(V,W)$ and $\mathcal{L}(V)$	Space formed by linear mappings from V to W; $\mathcal{L}(V)=\mathcal{L}(V,V)$
L	A linear mapping from operators to operators
$\mathscr{T}$	Choi isomorphism
I and $\mathcal{I}$	Identity operator and identity mapping
T and Γ	Transposition and partial transposition
P	A positive operator
H	A Hermitian operator
O	A generic operator
$\widetilde{H}^{\pm}$	H as a difference of positive operators
$H^{\pm}$	Like above, but by eigendecomposition
$\widetilde{L}_{\pm}$	L as the difference of two CP mappings
$L_{\pm}$	Like above, but by eigendecomposition
S and S_i	Quantum operations and sub-operations, unless explicitly specified
L^{Γ}	$\Gamma \circ L \circ \Gamma$
$S^{\Gamma}_{\pm}$	${S^{\Gamma}}_{\pm}$
$S^{\Gamma\dagger}_{i\ \pm}$	${{S_i}^{\Gamma}}_{\pm}{}^{\dagger}$
d_i	Dimension of Hilbert space $\mathcal{H}_i$
$\|\|\cdots\|\|$	Operator norm
$\|\|\cdots\|\|_p$	Schatten p-norm. When $p=1$ it's trace norm.
ker	The kernel or null space of a linear mapping
ran	The range/image of a mapping

Table 1: List of symbols and acronyms

$L^\dagger : \mathcal{L}(\mathcal{H}_2) \to \mathcal{L}(\mathcal{H}_1)$ is the adjoint of L. In particular, we're interested in $L^\dagger(I_2) \in \mathcal{L}(\mathcal{H}_1)$, because

$$\mathrm{tr}L(O_1) = (I_2|L(O_1)) = (L^\dagger(I_2)|O_1). \tag{5}$$

The trace of an operator after a linear mapping L can be expressed in terms of an inner product between the operator and $L^\dagger(I)$.

If L is HP, it always admits an operator-sum representation [11, 12, 13]:

$$L(O_1) = \sum_i c_i V_i O_1 V_i^\dagger,\ V_i \in \mathcal{L}(\mathcal{H}_1, \mathcal{H}_2) \text{ and } c_i \in \mathfrak{R}. \tag{6}$$

Therefore

$$L^\dagger(I) = \sum_i c_i V_i^\dagger V_i. \tag{7}$$

From this it's apparent $L^\dagger(I)$ is Hermitian if L is HP. A simple $L^\dagger(I)$ is more elegant and concise than the right side of (7), and that's one of the reasons why we express our results in, for example, $S_-^{\Gamma\dagger}(I)$, rather than the operators constituting the operator-sum representation of S^Γ.

Note [10]

$$(O_2 \otimes Q_2|O_1 \otimes Q_1) = (O_2|O_1)(Q_2|Q_1). \tag{8}$$

We will utilize this relation frequently for proofs.

1.4 Orthonormal basis of operators and Choi isomorphism

Consider a Hilbert space $\mathcal{H}_1$ with an orthonormal basis $\{a_i\}$. With Hilbert-Schmidt inner product, we can choose an orthonormal basis of operators for $\mathcal{L}(\mathcal{H}_1)$ [10, 14], by defining

$$E_{ij} := |a_i\rangle\langle a_j|. \tag{9}$$

Clearly $E_{ij}^\dagger = E_{ji}$, so $\{E_{ij}^\dagger\} = \{E_{ij}\}$.

Let $L : \mathcal{L}(\mathcal{H}_1) \to \mathcal{L}(\mathcal{H}_2)$. Choi isomorphism $\mathscr{T}$ then is defined as [12]

$$\mathscr{T}(L) := \mathcal{I} \otimes L(\sum_{i,j} E_{ij} \otimes E_{ij}) = \sum_{i,j} E_{ij} \otimes L(E_{ij}) \in \mathcal{L}(\mathcal{H}_1) \otimes \mathcal{L}(\mathcal{H}_2). \tag{10}$$

It can be found $L(O) = \mathrm{tr}_1(O^\mathrm{T} \otimes I\mathscr{T}(L))$ [15]:

$$\begin{aligned}
\mathrm{tr}_1(O^\mathrm{T} \otimes I\mathscr{T}(L)) &= \sum_{i,j} \mathrm{tr}(O^\mathrm{T} E_{ij}) L(E_{ij}) \\
&= \sum_{i,j} \mathrm{tr}(E_{ji} O) L(E_{ij}) \because \text{transposition is TP} \\
&= \sum_{i,j} (E_{ij}|O) L(E_{ij}) \\
&= L\Big(\sum_{i,j} E_{ij}(E_{ij}|O)\Big) \\
&= L(O).
\end{aligned}$$

The subscript beside tr refers to the party to be traced out; in this case, it's $\mathcal{H}_1$ (or $\mathcal{L}(\mathcal{H})$).

1.5 Bipartite system and partial transposition

When there are two parties Alice and Bob, let's consider a linear mapping from $\mathcal{L}(\mathcal{H}_{A_1} \otimes \mathcal{H}_{B_1})$ to $\mathcal{L}(\mathcal{H}_{A_2} \otimes \mathcal{H}_{B_2})$. We don't assume $\mathcal{H}_{A_1}$ and $\mathcal{H}_{A_2}$ are isomorphic, similarly for $\mathcal{H}_{B_1}$ and $\mathcal{H}_{B_2}$, so the dimensions before and after L can be different. Assign a basis for $\mathcal{L}(\mathcal{H}_{A_1})$ by (9), with $\{|a_i\rangle\}$ being an orthonormal basis of $\mathcal{H}_{A_1}$; then define a basis for $\mathcal{L}(\mathcal{H}_{B_1})$:

$$F_{ij} := |b_i\rangle\langle b_j|, \tag{11}$$

where $\{|bi\rangle\}$ is an orthonormal basis of B_1. Choi isomorphism for this composite system then is

$$\mathscr{T}(L) = \sum_{i,j,k,l} E_{ij} \otimes F_{kl} \otimes L(E_{ij} \otimes F_{kl}). \tag{12}$$

$\mathscr{T}(L)$ is in $\mathcal{L}(\mathcal{H}_{A_1}\otimes\mathcal{H}_{B_1}\otimes\mathcal{H}_{A_2}\otimes\mathcal{H}_{B_2})$. From now on $\{E_{ij}\}$ and $\{F_{ij}\}$ will denote bases of operators.

Transposition T is a linear mapping: $T:\mathcal{L}(H)\to\mathcal{L}(H)$. Similarly partial transposition, $\Gamma = T_A\otimes\mathcal{I}_B$, is also a linear mapping $\Gamma:\mathcal{L}(H_A\otimes H_B)\to\mathcal{L}(H_A\otimes H_B)$. They are both the inverses to themselves, i.e. $T\circ T=\mathcal{I}$ and $\Gamma\circ\Gamma=\mathcal{I}_{\mathcal{A}}\otimes\mathcal{I}_{\mathcal{B}}=\mathcal{I}_{\mathcal{AB}}$, so both are isomorphisms.

Partial transposition of a linear mapping L is defined as [16]

$$L^\Gamma := \Gamma\circ L\circ\Gamma. \tag{13}$$

Note for $L:\mathcal{L}(H_{A_1}\otimes H_{B_1})\to\mathcal{L}(H_{A_2}\otimes H_{B_2})$, the first (rightmost) Γ in (13) maps $\mathcal{L}(H_{A_1}\otimes H_{B_1})$ to $\mathcal{L}(H_{A_1}\otimes H_{B_1})$, and the second one maps $\mathcal{L}(H_{A_2}\otimes H_{B_2})$ to $\mathcal{L}(H_{A_2}\otimes H_{B_2})$. (13) itself is a linear mapping on linear mappings (on operators): If we define $f(L):=L^\Gamma$, then

$$f\in\mathcal{L}\left\{\mathcal{L}\left[\mathcal{L}(H_{A_1}\otimes H_{B_1}),\mathcal{L}(H_{A_2}\otimes H_{B_2})\right]\right\}.$$

Furthermore, as $(L^\Gamma)^\Gamma = L$, f is its own inverse, so it is also an isomorphism.

1.6 Trace, Schatten norm and positive operators

For P_1, $P_2\geq 0$, by the eigendecomposition $P_1=\sum_i p_i|\psi_i\rangle\langle\psi_i|$ we can find [6]:

$$\min_i p_i\mathrm{tr}P_2 \leq \mathrm{tr}P_1P_2 \leq \max_i p_i\mathrm{tr}P_2 = ||P_1||\,||P_2||_1. \tag{14}$$

where eigenvalue 0 is included here. The inequality on the right can be regarded as an application of Hölder's inequality [17]. Either side of (14) becomes an equality iff the subspace orthogonal to ker P_2 is in the eigensapce of P_1 with the maximum or minimum eigenvalue, e.g. if $P_1=pI$, $\mathrm{tr}P_1P_2=p||P_2||_1$. ker refers to the kernel or null space.

More generally, for an operator O its Schatten p-norm [9, 18, 19] is

$$||O||_p = \left(\mathrm{tr}|O|^p\right)^{1/p}, \tag{15}$$

where $|O|=\sqrt{O^\dagger O}$. For $p=\infty$ it is (defined as) the largest singular value of O, hence the same as the operator norm, $||\cdots||$ in our notation; for $p=1$ it is exactly the trace norm [18, 19]. Matrix Hölder inequality [20] states that for $1\leq p,q\leq\infty$ satisfying $1/p+1/q=1$:

$$|\mathrm{tr}O_1^\dagger O_2| = |(O_1,O_2)| \leq ||O_1||_p||O_2||_q. \tag{16}$$

As the upper bounds derived in our work are mainly based on (14), we can actually derive more general bounds using (16) and p- and q-norms, with $1/p+1/q=1$ and $1\leq p,q\leq\infty$. Further discussions will be given in Sec. 4.6.

From (14), we can easily see why for a sub-operation S_i,

$$0\leq S_i^\dagger(I)\leq I, \tag{17}$$

otherwise the probability could surpass 1 or be negative, and also why a TP mapping L should have $L^\dagger(I)=I$, which can also be regarded as a special case of the fact that $(O_1|O_2)=\mathrm{tr}O_2$ for all O_2 if and only if $O_1=I$. Additionally, the composition of TP mappings is still TP, so for a deterministic S

$$S^{\Gamma\dagger}(I)=I. \tag{18}$$

For any positive operator P, its trace is equal to its trace norm, $\mathrm{tr}P=||P||_1$. In this supplemental material (as in the main paper) we will use them interchangeably. As an example, for any CP mapping L, $L^\dagger(I)\geq 0$, so $\mathrm{tr}L^\dagger(I)=||L^\dagger(I)||_1$. More specifically, $S_{i\ \pm}^{\Gamma\dagger}(I)\geq 0$ and $\mathrm{tr}S_{i\ \pm}^{\Gamma\dagger}(I)=||S_{i\ \pm}^{\Gamma\dagger}(I)||_1$.

2 Hermitian operators: Decomposition and orthogonality

2.1 Decomposition of a Hermitian operator

Any Hermitian operator H can be decomposed as [21]

$$H=\widetilde{H}^+-\widetilde{H}^-,\ \widetilde{H}^\pm\geq 0. \tag{19}$$

Eigendecomposition $H = H^+ - H^-$ is one of such decompositions. Let ker denote the kernel or null space of an operator and ran the range or image of a mapping. Define

$$\mathcal{H}^\pm := (\ker H^\pm)^\perp = \mathrm{ran} H^\pm; \tag{20}$$

similarly for $\widetilde{\mathcal{H}}^\pm$. Hence,

$$\mathcal{H} = \mathcal{H}^+ \oplus \mathcal{H}^- \oplus \ker H, \tag{21}$$

and those three subspaces are mutually orthogonal.

Here is a lemma concerning such a decomposition, which is a generalization of Lemma 2 from Ref. [21]:

Lemma 1. *For any Hermitian operator H on a finite-dimensional Hilbert space $\mathcal{H}$, among all possible such decompositions: $H = \widetilde{H}^+ - \widetilde{H}^-$, $\widetilde{H}^\pm \geq 0$, the eigendecomposition $H = H^+ - H^-$ is the* ***unique*** *one that minimizes tr$\widetilde{H}^+$, tr$\widetilde{H}^-$, and tr$(\widetilde{H}^+ + \widetilde{H}^-)$; minimizing any one of them is the same as minimizing each of them. A decomposition where $\widetilde{\mathcal{H}}^\pm$ are orthogonal is equivalent to the eigendecomposition.*

Proof:

Eigendecomposition minimizes traces

We find $\mathcal{H}^\pm \subseteq \widetilde{\mathcal{H}}^\pm$; otherwise there would exist $|\psi^\pm\rangle \in \mathcal{H}^\pm$ such that $\langle\psi^\pm| H |\psi^\pm\rangle = \langle\psi^\pm| \widetilde{H}^+ - \widetilde{H}^- |\psi^\pm\rangle$ does not come out positive/negative. Furthermore, since $\widetilde{\mathcal{H}}^\pm$ may intersect $\mathcal{H}^\mp$ (and $\ker H$),

$$|\langle\psi^\pm| H |\psi^\pm\rangle| = \langle\psi^\pm| H^\pm |\psi^\pm\rangle \leq \langle\psi^\pm| \widetilde{H}^\pm |\psi^\pm\rangle, \tag{22}$$

because $\langle\psi^\pm| H |\psi^\pm\rangle = \langle\psi^\pm| \widetilde{H}^+ |\psi^\pm\rangle - \langle\psi^\pm| \widetilde{H}^- |\psi^\pm\rangle$ and $\widetilde{H}^\pm \geq 0$. Hence

$$\mathrm{tr}\widetilde{H}^\pm \geq \mathrm{tr}H^\pm, \tag{23}$$

i.e. eigendecomposition minimizes $\mathrm{tr}\widetilde{H}^\pm$ and $\mathrm{tr}\widetilde{H}^+ + \mathrm{tr}\widetilde{H}^-$.

Only eigendecomposition minimizes traces

Suppose we want to minimize both $\mathrm{tr}\widetilde{H}^\pm$ simultaneously. Because $\widetilde{H}^\pm \geq 0$ and because of (22), for both sides of (23) to be equal, i.e. to minimize both $\mathrm{tr}\widetilde{H}^\pm$, $\widetilde{\mathcal{H}}^\pm$ must be the same as $\mathcal{H}^\pm$ (as $\mathcal{H}^\pm \subseteq \widetilde{\mathcal{H}}^\pm$), implying

$$\pm\langle\psi^\pm| H |\phi^\pm\rangle = \langle\psi^\pm| \widetilde{H}^\pm |\phi^\pm\rangle = \langle\psi^\pm| H^\pm |\phi^\pm\rangle \tag{24}$$

and

$$\langle\psi^\pm| \widetilde{H} |\phi^\mp\rangle = 0, \tag{25}$$

where $|\psi^\pm\rangle$ and $|\phi^\pm\rangle$ are any vectors in $\mathcal{H}^\pm$; the same is true for any vectors in $\ker H$. Therefore $\langle\psi| \widetilde{H}^\pm |\phi\rangle = \langle\psi| H^\pm |\phi\rangle$ for any $|\phi\rangle, |\psi\rangle \in \mathcal{H}$ as $\mathcal{H} = \mathcal{H}^+ \oplus \mathcal{H}^- \oplus \ker H$, so $\widetilde{H}^\pm = H^\pm$. Because both $\mathrm{tr}\widetilde{H}^+$ and $\mathrm{tr}\widetilde{H}^-$ are minimized, so is $\mathrm{tr}(\widetilde{H}^+ + \widetilde{H}^-)$.

It is clear that to minimize $\mathrm{tr}(\widetilde{H}^+ + \widetilde{H}^-)$, both $\mathrm{tr}\widetilde{H}^+$ and $\mathrm{tr}\widetilde{H}^-$ should be minimized, leading to the same conclusion. If we just want to minimize one of $\mathrm{tr}\widetilde{H}^+$ and $\mathrm{tr}\widetilde{H}^-$, say $\mathrm{tr}\widetilde{H}^-$, then by (23) $\mathrm{tr}\widetilde{H}^- = \mathrm{tr}H^-$. Because $\mathrm{tr}H = \mathrm{tr}H^+ - \mathrm{tr}H^- = \mathrm{tr}\widetilde{H}^+ - \mathrm{tr}\widetilde{H}^-$, $\mathrm{tr}\widetilde{H}^+ = \mathrm{tr}H^+$, i.e. both $\mathrm{tr}\widetilde{H}^+$ and $\mathrm{tr}\widetilde{H}^-$ are minimized. Hence minimizing any of them is identical to minimizing all of them, and minimization of traces requires eigendecomposition.

Orthogonality is equivalent to eigendecomposition

It has been known that if $\widetilde{H}^\pm = H^\pm$, $\widetilde{\mathcal{H}}^+ \perp \widetilde{\mathcal{H}}^-$, so we only have to show the reverse. If $\widetilde{\mathcal{H}}^+ \perp \widetilde{\mathcal{H}}^-$, because $\mathcal{H}^\pm \subseteq \widetilde{\mathcal{H}}^\pm$ and $\mathcal{H}^+ \perp \mathcal{H}^-$, $\widetilde{\mathcal{H}}^\pm \subseteq \mathcal{H}^\pm \oplus \ker H$. For any vector $|\psi\rangle \in \ker H$, $\widetilde{H}^+|\psi\rangle = \widetilde{H}^-|\psi\rangle$; however, because $\widetilde{\mathcal{H}}^+ \perp \widetilde{\mathcal{H}}^-$, $\widetilde{H}^+|\psi\rangle = \widetilde{H}^-|\psi\rangle = 0$, i.e. $\ker H \subseteq \ker \widetilde{H}^\pm$, which, by $\widetilde{\mathcal{H}}^\pm \subseteq \mathcal{H}^\pm \oplus \ker H$, implies $\widetilde{\mathcal{H}}^\pm \subseteq \mathcal{H}^\pm$, as $\ker \widetilde{H}^\pm \perp \widetilde{\mathcal{H}}^\pm$. Since $\mathcal{H}^\pm \subseteq \widetilde{\mathcal{H}}^\pm$, we conclude $\mathcal{H}^\pm = \widetilde{\mathcal{H}}^\pm$, so according to the arguments from the second part of the proof $\widetilde{H}^\pm = H^\pm$.

Or alternatively, we can directly apply the spectral theorem [22]: As $\widetilde{H}^\pm$ are orthogonal, (after eigendecomposing $\widetilde{H}^\pm$) $\widetilde{H}^+ - \widetilde{H}^-$ is an orthogonal spectral resolution [22] of H.

□

2.1.1 How to construct such a decomposition

In this section we will discuss given a Hermitian operator H, how to construct such a decomposition for it. The goal is to find a Hermitian operator H' such that

$$\widetilde{H}^{\pm} = H^{\pm} + H' \geq 0. \tag{26}$$

From (22) and $\mathcal{H}^{\pm} \subseteq \widetilde{\mathcal{H}}^{\pm}$ it may seem $\widetilde{H}^{\pm} \geq H^{\pm}$, but this is not true in general—what (22) implies is actually $\widetilde{H}^{\pm} \geq H^{\pm}$ *to the restriction of* $\mathcal{H}^{\pm}$. To be specific, for a vector $|\psi^{+}\rangle + |\psi^{-}\rangle$ where $|\psi^{\pm}\rangle \in \mathcal{H}^{\pm}$, the value of

$$\left(\langle\psi^{+}| + \langle\psi^{-}|\right) H' \left(|\psi^{+}\rangle + |\psi^{-}\rangle\right), \tag{27}$$

even though $\langle\psi^{\pm}| H' |\psi^{\pm}\rangle \geq 0$, is not necessarily non-negative, as it depends on $\langle\psi^{\pm}| H' |\psi^{\mp}\rangle$ as well. In terms of matrices, it just reflects the fact that *a matrix that has non-negative diagonal entries are not necessarily positive (semi-definite).*

Let's choose a random orthonormal basis $\{|\phi_i\rangle\}$, and use it to construct H': $\sum_i a_i |\phi_i\rangle\langle\phi_i|$. The eigenvalues a_i are to be determined. Defining matrices M and A as

$$M_{ij} := \langle\phi_i| H' |\phi_i\rangle \text{ and } A_{ij} := a_i \delta_{ij}, \tag{28}$$

(26) is equivalent to

$$M - A \geq 0. \tag{29}$$

Now we can try different combination of $\{a_i\}$; if (29) is satisfied, then we find a valid decomposition. Sylvester's theorem may be useful in this process.

The benefit of choosing the eigenvectors of H' as the standard basis is that we have less parameters to fix. Here are some guidelines on how to select the basis and eigenvalues:

1. $\mathrm{tr} H' \geq 0$, as Lemma 1 suggests, i.e. $\sum_i a_i \geq 0$.
2. For $a_i < 0$, the corresponding $|\phi_i\rangle \notin \ker H$ and $|\phi_i\rangle \notin \mathcal{H}^{\pm}$. The reason is that, if $|\phi_i\rangle \in \mathcal{H}^{+}$, then $|\phi_i\rangle \perp \mathcal{H}^{-}$ and $\langle\phi_i| H^{-} + H' |\phi_i\rangle = a_i < 0$.
3. As a positive operator is also positive to the restriction of any subspace, it is advisable to determine the negative parts of H' first, and check if $H^{\pm} + H'$ is positive when restricted to the subspace $\mathcal{H}'^{-}$; however it should be emphasized even if $H^{\pm} + H' \geq 0$ to the restriction to this subspace, it does not mean $H^{\pm} + H' \geq 0$ over the entire space.
4. Similarly, for $M - A \geq 0$ it is necessary that $M - A$ have non-negative diagonal elements, but it is not a sufficient condition, as we have pointed out. The third and fourth points can be regarded as an application of Sylvester's theorem.

2.2 Orthogonality of ensembles

Lemma 2. *Suppose P_1 and P_2 are two positive operators, and they have such ensembles:[23, 24]*

$$P_i = \sum_j |\psi_j^i\rangle\langle\psi_j^i|, \tag{30}$$

where each $\{|\psi_j^i\rangle\}$ is a set of nonzero vectors that aren't necessarily normalized or mutually orthogonal. Then each eigenvector/eigenspace of P_2 corresponding to a nonzero eigenvalue is orthogonal to each of P_1 if and only if

$$\langle\psi_i^1|\psi_j^2\rangle = 0 \; \forall i, j,$$

in other words, if and only if

$$\mathrm{ran} P_1 \perp \mathrm{ran} P_2. \tag{31}$$

Proof. From Ref. [23], if the eigenensembles of P_i are

$$P_i = \sum_{j(i)} |e_j^i\rangle\langle e_j^i|, \tag{32}$$

where $|e^i_j\rangle$ aren't normalized, then there exist unitary matrices U^i such that

$$\begin{cases} |e^i_j\rangle = \sum_k U^i_{jk}|\psi^i_k\rangle & j \leq \text{Rank}(P_i) \\ |0\rangle = \sum_k U^i_{jk}|\psi^i_k\rangle & j > \text{Rank}(P_i) \end{cases}, \tag{33}$$

and

$$|\psi^i_j\rangle = \sum_k (U^i)^{-1}_{jk}|e^i_k\rangle. \tag{34}$$

If $\langle e^1_i|e^2_j\rangle = 0\ \forall i,j$, from (34) we can see $\langle\psi^1_i|\psi^2_j\rangle = 0\ \forall i,j$. Similarly, if $\langle\psi^1_i|\psi^2_j\rangle = 0$ then from (33) $\langle e^1_i|e^2_j\rangle = 0$ $\forall i,j$. In other words, because each $|\psi^i_j\rangle$ is a linear combination of $\{|e^i_j\rangle\}$, and each $|e^i_j\rangle$ is also linear combinations of $\{|\psi^i_j\rangle\}$, orthogonality of two ensembles guarantees that of the other two.

Here's a more elegant proof: The orthogonality of the vectors as stated in the lemma is satisfied if and only if their spans are orthogonal. From (32), the spans of $\{\psi^i_j\}$ are identical to $(\ker P_i)^\perp = \text{ran}P_i$, which of course are also identical to the spans of $\{e^i_j\}$. Therefore the orthogonality of one pair implies that of the other. □

3 Adjoint of a linear mapping and complex conjugation of an operator

3.1 Adjoint

With $L : \mathcal{L}(\mathcal{H}_1) \to \mathcal{L}(\mathcal{H}_2)$, $\mathscr{T}(L) \in \mathcal{L}(\mathcal{H}_1) \otimes \mathcal{L}(\mathcal{H}_2)$. Hence tr_2 in the following lemma traces out $\mathcal{L}(\mathcal{H}_2)$.

Lemma 3. *For a linear mapping L,*

$$L^\dagger(I) = tr_2\mathscr{T}(L)^*.$$

Proof. As in Sec. 1.4, we can find

$$\begin{aligned} \text{tr}L(O) &= \text{tr}(O^{\text{T}} \otimes I\mathscr{T}(L)) \\ &= \sum_{i,j} \text{tr}(O^{\text{T}}E_{ij})\text{tr}L(E_{ij}) \\ &= \sum_{i,j} (E_{ij}|O)\text{tr}L(E_{ij}) \\ &= \Big(\sum_{i,j} E_{ij}\text{tr}L(E_{ij})^*\Big|O\Big), \end{aligned} \tag{35}$$

where $L(E_{ij})^* = (L(E_{ij}))^*$. In addition, since $E^*_{ij} = E_{ij}$,

$$\text{tr}_2\mathscr{T}(L)^* = \sum_{i,j} E_{ij}\text{tr}L(E_{ij})^*. \tag{36}$$

Because

$$\text{tr}L(O) = (I|L(O)) = (L^\dagger(I)|O),$$

by comparing (35) and (36) we can conclude $L^\dagger(I) = \text{tr}_2\mathscr{T}(L)^*$. □

3.2 Complex conjugation and basis

Note the importance of the basis for $\mathcal{H}_1$ chosen in the proof above, because the transpose and complex conjugation of an operator depend on the basis. As for $\mathcal{H}_2$, since $\text{tr}(A^*) = (\text{tr}A)^*$ with whichever (orthonormal) basis, it does not matter.

Now assume the mapping L is HP, so we can perform a spectral decomposition on $\mathscr{T}(L)$. Suppose its eigendecomposition is $\mathscr{T}(L) = \sum_i c_i|v_i\rangle\langle v_i|$, with

$$|v_i\rangle = \sum_{j,k} d^i_{jk}|a_j\rangle|b_k\rangle,$$

where $\{b_i\}$ is any orthonormal basis of $\mathcal{H}_2$, and $E_{ij} = |a_i\rangle\langle a_j|$ is the basis chosen for Choi isomorphism. Then,

$$\begin{aligned}(|v_i\rangle\langle v_i|)^* &= \sum_{j,k,l,m} {d^i_{jk}}^* d^i_{lm} |a_j\rangle\langle a_l| \otimes |b_k\rangle\langle b_m| \\ &= \sum_{j,k} {d^i_{jk}}^* |a_j\rangle|b_k\rangle \sum_{l,m} d^i_{lm} \langle a_l|\langle b_m| \\ &= |v_i^*\rangle\langle v_i^*|, \end{aligned} \tag{37}$$

where

$$|v_i^*\rangle := \sum_{j,k} {d^i_{jk}}^* |a_j\rangle|b_k\rangle.$$

Because $\langle u|v\rangle^* = \langle u^*|v^*\rangle$,

$$\langle v_i^*|v_j^*\rangle = \langle v_i|v_j\rangle^* = \delta_{ij}. \tag{38}$$

We thus obtain

$$\mathscr{T}(L)^* = \sum_i c_i |v_i^*\rangle\langle v_i^*|, \tag{39}$$

and $\{|v_i^*\rangle\}$ is an orthonormal basis. For a vector $|v\rangle \in \mathcal{H}_1 \otimes \mathcal{H}_2$, after fixing the basis for $\mathcal{H}_1$, $|v^*\rangle$ depends on the basis for $\mathcal{H}_2$, but $\mathrm{tr}_2|v^*\rangle\langle v^*|$ would be the same regardless of the basis for $\mathcal{H}_2$.

From (38) and (39), $\mathscr{T}(L)^*$ and $\mathscr{T}(L)$ have the same eigenvalues, so

Corollary 1. *For any HP mapping L,*

$$trL^\dagger(I) = tr\mathscr{T}(L). \tag{40}$$

4 Proof of Proposition 1 and 2

Let's restate Proposition 1 from the main article:

Proposition 1. *1. There exist upper and lower bounds for entangling capacities of deterministic operations:*

$$\begin{aligned} \frac{||{S^\Gamma_-}^\dagger(I)||_1}{d_A d_B} &\le EC_N(S) \le ||{\widetilde{S^\Gamma}_-}^\dagger(I)||\, ||\rho^\Gamma||_1 \\ \log \frac{||\mathscr{T}(S^\Gamma)||_1}{d_A d_B} = \log\left(1 + 2\frac{||{S^\Gamma_-}^\dagger(I)||_1}{d_A d_B}\right) &\le EC_L(S) \le \log(1 + 2||{\widetilde{S^\Gamma}_-}^\dagger(I)||). \end{aligned} \tag{41}$$

2. For a probabilistic operation composed of sub-operations S_i,

$$\begin{aligned} \sum_i \frac{||{S^\Gamma_{i-}}^\dagger(I)||_1}{d_A d_B} &\le EC_N(S) \le ||\sum_i {\widetilde{S^\Gamma_i}_-}^\dagger(I)||\, ||\rho^\Gamma||_1, \\ \sum_i \frac{tr\mathscr{T}(S_i)}{d_A d_B} \log \frac{||\mathscr{T}(S_i^\Gamma)||_1}{tr\mathscr{T}(S_i)} &\le EC_L(S) \le \log(1 + 2||\sum_i {\widetilde{S^\Gamma_i}_-}^\dagger(I)||). \end{aligned}$$

The upper bounds of part 2 can be applied to a deterministic operation $S = \sum_i S_i$.

3. With an initial negativity E_N, the expected negativity, i.e. probability times p_i the actual negativity E_{Ni}, after a sub-operation S_i is bounded by:

$$p_i E_{Ni} \le E_N\left(||{\widetilde{S^\Gamma_i}_-}^\dagger(I)|| + ||{\widetilde{S^\Gamma_i}_+}^\dagger(I)||\right) + ||{\widetilde{S^\Gamma_i}_-}^\dagger(I)||.$$

The entangling capacity of a sub-operation is bounded from below by:

$$\begin{aligned} EC_N(S_i) &\ge \frac{tr{S^\Gamma_{i-}}^\dagger(I)}{trS_i^\dagger(I)} = \left(\frac{tr{S^\Gamma_{i+}}^\dagger(I)}{tr{S^\Gamma_{i-}}^\dagger(I)} - 1\right)^{-1}, \\ EC_L(S_i) &\ge \log\left(1 + 2\frac{tr{S^\Gamma_{i-}}^\dagger(I)}{trS_i^\dagger(I)}\right) = \log\left[1 + 2\left(\frac{tr{S^\Gamma_{i+}}^\dagger(I)}{tr{S^\Gamma_{i-}}^\dagger(I)} - 1\right)^{-1}\right] \end{aligned}$$

All the upper bounds remain the same after the addition of an ancilla: $||(\mathcal{I} \otimes \widetilde{S^\Gamma}_-)^\dagger(I)|| = ||{\widetilde{S^\Gamma_i}_-}^\dagger(I)||$.

4.1 Some equalities

Here are some relevant equalities. Suppose $L : \mathcal{L}(\mathcal{H}_1) \to \mathcal{L}(\mathcal{H}_2)$, and the dimension of $\mathcal{H}_1$ is d.

If L is TP,

$$\mathrm{tr}\mathscr{T}(L) = \mathrm{tr}\sum_{i,j} E_{ij} \otimes L(E_{ij}) = \sum_{ij} \delta_{ij}^2 = d. \tag{42}$$

Therefore for a TP operation S, $\mathrm{tr}\mathscr{T}(S) = \mathrm{tr}\mathscr{T}(S^\Gamma) = d_A d_B$. Note S^Γ is also TP.

If L is HPTP, then $L^\dagger(I) = \widetilde{L}_+^\dagger(I) - \widetilde{L}_-^\dagger(I) = I$, so $\widetilde{L}_+^\dagger(I) = I + \widetilde{L}_-^\dagger(I)$. Hence

$$1 + 2||\widetilde{L}_-^\dagger(I)|| = ||I + 2\widetilde{L}_-^\dagger(I)|| = ||\widetilde{L}_+^\dagger(I) + \widetilde{L}_-^\dagger(I)||. \tag{43}$$

because $||I + P|| = 1 + ||P||$ for $P \geq 0$, and

$$d + 2||\widetilde{L}_-^\dagger(I)||_1 = ||I + 2\widetilde{L}_-^\dagger(I)||_1 = ||\widetilde{L}_+^\dagger(I) + \widetilde{L}_-^\dagger(I)||_1. \tag{44}$$

4.2 Upper bounds

We start with

Lemma 4. *For an HPTP mapping L and Hermitian H,*

$$trL(H)^- - trH^- \leq ||\widetilde{L}_-^\dagger(I)||\,||H||_1,$$
$$||L(H)||_1 - ||H||_1 \leq 2||\widetilde{L}_-^\dagger(I)||\,||H||_1.$$

Proof. For any Hermitian H [21]

$$||H||_1 = \mathrm{tr}H^+ + \mathrm{tr}H^- = \mathrm{tr}H + 2\mathrm{tr}H^-. \tag{45}$$

With an HP L, since $\widetilde{L}_\pm$ are CP, by Lemma 1 [6]

$$\mathrm{tr}L(H)^\pm \leq \mathrm{tr}\widetilde{L}_+(H^\pm) + \mathrm{tr}\widetilde{L}_-(H^\mp). \tag{46}$$

Since L is TP as well,

$$\begin{aligned}\mathrm{tr}L(H)^- - \mathrm{tr}H^- &\leq \mathrm{tr}\left[\widetilde{L}_+(H^-) + \widetilde{L}_-(H^+) - L(H^-)\right] \\ &= \mathrm{tr}\left[\widetilde{L}_-^\dagger(I)(H^+ + H^-)\right] \\ &\leq ||\widetilde{L}_-^\dagger(I)||\,||H||_1 \text{ by (14)}.\end{aligned}$$

By (45) and L being TP,

$$\begin{aligned}||L(H)||_1 - ||H||_1 &= 2(\mathrm{tr}L(H)^- - \mathrm{tr}H^-) \\ &\leq 2||\widetilde{L}_-^\dagger(I)||\,||H||_1.\end{aligned}$$

The proof is completed. □

4.2.1 Deterministic operation

An operation S is CP, so it is HP [2, 11, 12]. Because Γ is HPTP, S^Γ is HPTP. Hence we can apply Lemma 4 to $S^\Gamma(\rho^\Gamma) = S(\rho)^\Gamma$:

$$\begin{aligned}E_N(S(\rho)) - E_N(\rho) &= \mathrm{tr}S^\Gamma(\rho^\Gamma)^- - \mathrm{tr}\rho^{\Gamma-} \\ &\leq ||\widetilde{S^\Gamma}^\dagger_-(I)||\,||\rho^\Gamma||_1,\end{aligned} \tag{47}$$

and

$$\begin{aligned}E_L(S(\rho)) - E_L(\rho) &= \log||S^\Gamma(\rho^\Gamma)||_1 - \log||\rho^\Gamma||_1 \\ &= \log(1 + \frac{||S^\Gamma(\rho^\Gamma)||_1 - ||\rho^\Gamma||_1}{||\rho^\Gamma||_1}) \\ &\leq \log(1 + 2||\widetilde{S^\Gamma}^\dagger_-(I)||).\end{aligned} \tag{48}$$

The upper bounds from part 1 of Proposition 1 are proven.

For $L=\sum_i L_i$, where L_i are HP and L is TP, we can choose $\widetilde{L}_\pm = \sum_i \widetilde{L_{i\pm}}$. With

$$(\sum_i \widetilde{L_{i-}})^\dagger = \sum_i \widetilde{L_{i-}}^\dagger,$$

by Lemma 4 we obtain

$$\mathrm{tr} L(H)^- - \mathrm{tr} H^- \leq ||\sum_i \widetilde{L_{i-}}^\dagger (I)||\, ||H||_1$$

by the triangle inequality. Therefore for a deterministic operation $S=\sum_i S_i$, if we choose $\widetilde{S^\Gamma}_\pm = \sum_i (\widetilde{S_i^\Gamma}_\pm)$,

$$E_N(S(\rho)) - E_N(\rho) \leq ||\sum_i \widetilde{S_i^\Gamma}_-^\dagger (I)||\, ||\rho^\Gamma||_1 \tag{49}$$

and

$$E_L(S(\rho)) - E_L(\rho) \leq \log(1+2||\sum_i \widetilde{S_i^\Gamma}_-^\dagger (I)||) \tag{50}$$

Hence we have proved part 2 of Proposition 1 can be applied to deterministic $S=\sum_i S_i$.

Let's emphasize again $\widetilde{S^\Gamma}_\pm = \sum_i(\widetilde{S_i^\Gamma}_\pm)$ are some of the possible decompositions of S^Γ, and they may or may not be the ideal choices that yield the smallest bounds.

4.2.2 Probabilistic operation

Let's prove the upper bounds from part 2 of Proposition 1 for probabilistic operations. The average negativity after S is

$$\sum_i p_i E_{N i} = \sum_i p_i \frac{\mathrm{tr} S_i(\rho)^{\Gamma -}}{p_i} = \sum_i \mathrm{tr} S_i(\rho)^{\Gamma -}. \tag{51}$$

Furthermore, by (46)

$$\begin{aligned}\sum_i \mathrm{tr} S_i(\rho)^{\Gamma\pm} &\leq \sum_i [\mathrm{tr}\widetilde{S_i^\Gamma}_+(\rho^{\Gamma\pm}) + \mathrm{tr}\widetilde{S_i^\Gamma}_-(\rho^{\Gamma\mp})] \\ &= \mathrm{tr}\left(\sum_i \widetilde{S_i^\Gamma}_+\right)(\rho^{\Gamma\pm}) + \mathrm{tr}\left(\sum_i \widetilde{S_i^\Gamma}_-\right)(\rho^{\Gamma\mp}).\end{aligned} \tag{52}$$

Essentially, we now have $S^\Gamma = \sum_i S_i^\Gamma = \sum_i \widetilde{S_i^\Gamma}_+ - \sum_i \widetilde{S_i^\Gamma}_-$, with $\widetilde{S^\Gamma}_\pm = \sum_i \widetilde{S_i^\Gamma}_\pm$, and S is TP as in the deterministic case, c.f. (46). Thus Lemma 4 can be used, and we essentially recover the right sides of (49) for the bounds of $\sum_i p_i E_{N i} - E_N$, where E_N is the initial negativity.

As to the bounds for EC_L of a probabilistic operation, with an initial logarithmic negativity E_L:

$$\begin{aligned}\sum p_i E_{L i} - E_L &= \sum_i p_i \log ||\rho_i^\Gamma||_1 - \log ||\rho^\Gamma||_1 \\ &= \sum_i p_i \log \frac{||S_i^\Gamma(\rho^\Gamma)||_1}{p_i} - \log ||\rho^\Gamma||_1 \\ &\leq \log \sum_i ||S_i^\Gamma(\rho^\Gamma)||_1 - \log ||\rho^\Gamma||_1 \\ &= \log\left(1 + \frac{\sum_i ||S_i^\Gamma(\rho^\Gamma)||_1 - ||\rho^\Gamma||_1}{||\rho^\Gamma||_1}\right),\end{aligned} \tag{53}$$

by the concavity of logarithm and Jensen's inequality [17, 25, 26]. By (45),

$$\begin{aligned}\sum_i ||S_i^\Gamma(\rho^\Gamma)||_1 &= \sum_i \left[\mathrm{tr} S_i^\Gamma(\rho^\Gamma) + 2\mathrm{tr} S_i^\Gamma(\rho^\Gamma)^-\right] \\ &= \mathrm{tr}\sum_i S_i^\Gamma(\rho^\Gamma) + 2\sum_i \mathrm{tr} S_i^\Gamma(\rho^\Gamma)^- \\ &= \mathrm{tr}\rho^\Gamma + 2\sum_i \mathrm{tr} S_i^\Gamma(\rho^\Gamma)^-,\end{aligned} \tag{54}$$

because $S^\Gamma = \sum_i S_i^\Gamma$ is TP. Now we are back to (52). Following the same steps as before, we can obtain the desired result.

4.2.3 Sub-operation

Let's move on to upper bounds from part 3 of Proposition 1. By (14), (45) and (46),

$$\begin{aligned}p_i E_{N\,i} = \mathrm{tr} S_i^\Gamma(\rho^\Gamma) &\leq ||\widetilde{S_i^\Gamma}_+^\dagger(I)||\,|||\rho^{\Gamma^-}||_1 + ||\widetilde{S_i^\Gamma}_-^\dagger(I)||\,||\rho^{\Gamma^+}||_1 \\ &= ||\widetilde{S_i^\Gamma}_+^\dagger(I)||\,||\rho^{\Gamma^-}||_1 + ||\widetilde{S_i^\Gamma}_-^\dagger(I)||\left(1 + ||\rho^{\Gamma^-}||_1\right) \\ &= E_N\left(||\widetilde{S_i^\Gamma}_+^\dagger(I)|| + ||\widetilde{S_i^\Gamma}_-^\dagger(I)||\right) + ||\widetilde{S_i^\Gamma}_-^\dagger(I)||.\end{aligned}$$

4.3 Lower bounds

4.3.1 Deterministic operation

Employing the method proposed by Campbell [6], to obtain the lower bounds for entangling capacities, an ancilla is used: We apply the operation $\mathcal{I}_{A_aB_a} \otimes S_{A_sB_s}$ on the state $|\Psi\rangle$ defined as:

$$\begin{aligned}|\Psi\rangle\langle\Psi| :=& \frac{1}{d_A d_B}\sum_{i,j,k,l} |a_i\rangle\langle a_j| \otimes |b_k\rangle\langle b_l| \otimes |a_i\rangle\langle a_j| \otimes |b_k\rangle\langle b_l| \\ =& |\Psi_{A_aA_s}\rangle\langle\Psi_{A_aA_s}| \otimes |\Psi_{B_aB_s}\rangle\langle\Psi_{B_aB_s}|, \\ =& \frac{1}{d_A d_B}\sum_{i,j,k,l} E_{ij}\otimes F_{kl}\otimes E_{ij}\otimes F_{kl} \end{aligned} \tag{55}$$

where

$$|\Psi_{A_aA_s}\rangle := \frac{1}{\sqrt{d_A}}|a_i\rangle|a_i\rangle \tag{56}$$

is a maximally entangled state, similarly for $|\Psi_{B_aB_s}\rangle$. Here A_a and B_a are the ancilla, and are clones of the original system A_s and B_s (By clones we mean $\mathcal{H}_{A_a}$ and $\mathcal{H}_{B_a}$ are isomorphic to $\mathcal{H}_{A_s}$ and $\mathcal{H}_{B_s}$. An ancilla in general does not necessarily have to be isomorphic to the system). (55) is essentially a normalized Choi isomorphism.

We will ignore the subscripts of $\mathcal{I}_{A_aB_a}$ and $S_{A_sB_s}$ from now on. As A and B are not entangled,

$$\mathcal{I}\otimes S|\Psi\rangle\langle\Psi| = \mathscr{T}(S)/(d_A d_B) \tag{57}$$

alone can give us a lower bound for entangling capacity. With $\Gamma = T_A \otimes \mathcal{I}_B$, we have

$$\begin{aligned}\mathscr{T}(S)^\Gamma &= d_A d_B\left(\mathcal{I}\otimes S|\Psi\rangle\langle\Psi|\right)^\Gamma \\ &= \mathcal{I}\otimes S^\Gamma\Big(\sum_{i,j,k,l} E_{ij}\otimes F_{kl}\otimes E_{ij}\otimes F_{kl}\Big)^\Gamma \\ &= \mathcal{I}\otimes S^\Gamma\Big(\sum_{i,j,k,l} E_{ji}\otimes F_{kl}\otimes E_{ji}\otimes F_{kl}\Big) \\ &= \mathscr{T}(S^\Gamma),\end{aligned} \tag{58}$$

because $(\mathcal{I}\otimes S)^\Gamma = \mathcal{I}^\Gamma \otimes S^\Gamma = \mathcal{I}\otimes S^\Gamma$. Therefore

$$\left[\mathscr{T}(S)^\Gamma\right]^\pm = \mathscr{T}(S^\Gamma)^\pm = \mathscr{T}(S^\Gamma_\pm). \tag{59}$$

By Corollary 1, $\mathrm{tr}\mathscr{T}(S^\Gamma_\pm) = \mathrm{tr}{S^\Gamma_\pm}^\dagger(I)$, so

$$\mathrm{tr}\left\{\left[\mathcal{I}\otimes S(|\Psi\rangle\langle\Psi|)\right]^\Gamma\right\}^\pm = \frac{1}{d_A d_B}\mathrm{tr}\mathscr{T}(S^\Gamma_\pm) = \frac{1}{d_A d_B}\mathrm{tr}{S^\Gamma_\pm}^\dagger(I). \tag{60}$$

Alternatively, we can directly calculate $\mathrm{tr}\mathscr{T}(S^\Gamma_\pm)$ without using Corollary 1:

$$\begin{aligned}\mathrm{tr}\mathscr{T}(S^\Gamma_\pm) &= \mathrm{tr}\sum_{i,j,k,l} E_{ji}\otimes F_{kl}\otimes S^\Gamma_\pm(E_{ij}\otimes F_{kl}) \\ &= \sum_{i,j,k,l}\delta_{ij}\delta_{kl}\mathrm{tr}S^\Gamma_\pm(E_{ij}\otimes F_{kl}) \\ &= \mathrm{tr}S^\Gamma_\pm\left(\sum_{i,k} E_{ii}\otimes F_{kk}\right) \\ &= \mathrm{tr}{S^\Gamma_\pm}^\dagger(I), \end{aligned} \tag{61}$$

because $\mathrm{tr}L(I) = (L^\dagger(I)|I) = \mathrm{tr}(L^\dagger(I))^\dagger$, and $L^\dagger(I)$ is Hermitian is L is HP, (7). Hence

$$E_N(\mathcal{I}\otimes S(|\Psi\rangle\langle\Psi|)) = \frac{1}{d_Ad_B}\mathrm{tr}\mathscr{T}(S^\Gamma_-) = \frac{1}{d_Ad_B}\mathrm{tr}{S^\Gamma_-}^\dagger(I).$$

We can use (45) to acquire the bound for logarithmic negativity as in Proposition 1. Or, because $\mathscr{T}(S^\Gamma_\pm)$ are orthogonal, by (60)

$$||\left[\mathcal{I}\otimes S(|\Psi\rangle\langle\Psi|)\right]^\Gamma||_1 = \frac{1}{d_Ad_B}||\mathscr{T}(S^\Gamma)||_1, \tag{62}$$

leading to one of the expressions of the lower bound. As $||\mathscr{T}(S^\Gamma)||_1 = \mathrm{tr}\mathscr{T}(S^\Gamma_+) + \mathrm{tr}\mathscr{T}(S^\Gamma_-)$, by Corollary 1,

$$||\mathscr{T}(S^\Gamma)||_1 = \mathrm{tr}{S^\Gamma_+}^\dagger(I) + \mathrm{tr}{S^\Gamma_-}^\dagger(I). \tag{63}$$

Since ${S^\Gamma_+}^\dagger(I) - {S^\Gamma_-}^\dagger(I) = I$,

$$\mathrm{tr}{S^\Gamma_+}^\dagger(I) + \mathrm{tr}{S^\Gamma_-}^\dagger(I) = \mathrm{tr}I + 2\mathrm{tr}{S^\Gamma_-}^\dagger(I) = d_Ad_B + 2\mathrm{tr}{S^\Gamma_-}^\dagger(I), \tag{64}$$

which gives us another expression of the lower bound. We can also use (44) to show this relation.

4.3.2 Sub-operation

To obtain the lower bounds for a sub-operation the procedures are pretty much the same, but now we should take the probability into account:

$$p_i = \mathrm{tr}\mathcal{I}\otimes S_i(|\Psi\rangle\langle\Psi|) = \frac{\mathrm{tr}\mathscr{T}(S_i)}{d_Ad_B} = \frac{\mathrm{tr}S_i^\dagger(I)}{d_Ad_B}, \tag{65}$$

c.f. (60) or (61). Use the same method to obtain $\mathrm{tr}\mathcal{I}\otimes S^\Gamma_{i\,-}(|\Psi\rangle\langle\Psi|)$. Thus the negativity is

$$E_{N\,i}(|\Psi\rangle\langle\Psi|) = \frac{\mathrm{tr}{S^\Gamma_{i\,-}}^\dagger(I)}{\mathrm{tr}S_i^\dagger(I)}. \tag{66}$$

Similarly,

$$||\mathcal{I}\otimes S_i(|\Psi\rangle)\langle\Psi|||_1 = \frac{||\mathscr{T}(S^\Gamma_i)||_1}{d_Ad_B} = \frac{\mathrm{tr}{S^\Gamma_{i\,+}}^\dagger(I) + \mathrm{tr}{S^\Gamma_{i\,-}}^\dagger(I)}{d_Ad_B}, \tag{67}$$

c.f. (63), so

$$E_{L\,i}(|\Psi\rangle\langle\Psi|) = \log\frac{||\mathscr{T}(S^\Gamma_i)||_1}{\mathrm{tr}S_i^\dagger(I)} = \log\frac{\mathrm{tr}{S^\Gamma_{i\,+}}^\dagger(I) + \mathrm{tr}{S^\Gamma_{i\,-}}^\dagger(I)}{\mathrm{tr}S_i^\dagger(I)}. \tag{68}$$

The lower bounds for deterministic operations can be regarded as a special case of sub-operations—this can be easily shown with Corollary 1 and (42).

Because transposition and partial transposition are TP, $\mathrm{tr}\mathscr{T}(L) = \mathrm{tr}\mathscr{T}(L^\Gamma)$. For an HP L, it means

$$\mathrm{tr}\mathscr{T}(L) = \mathrm{tr}\mathscr{T}(L^\Gamma) = \mathrm{tr}\mathscr{T}(L^\Gamma_+) - \mathrm{tr}\mathscr{T}(L^\Gamma_-).$$

Therefore by Corollary 1

$$\mathrm{tr}{S_i}^\dagger(I) = \mathrm{tr}{S_i^\Gamma}^\dagger(I) = \mathrm{tr}{S^\Gamma_{i\,+}}^\dagger(I) - \mathrm{tr}{S^\Gamma_{i\,-}}^\dagger(I). \tag{69}$$

With (69) we can adjust (66) and (68) to our liking, such as in Proposition 1. Note in general we can't expect ${S_i^\Gamma}^\dagger(I) \geq 0$, so its trace may not equate to its trace norm.

4.3.3 Probabilistic operation

For the average negativity of a probabilistic operation, by (65) and (66)

$$\sum_i p_i E_N(\mathcal{I}\otimes S_i(|\Psi\rangle\langle\Psi|)) = \sum_i \frac{\mathrm{tr} {S_i^\Gamma}^\dagger_-(I)}{d_A d_B}. \tag{70}$$

For the average logarithmic negativity, by (65) and (68)

$$\sum_i p_i E_L(\mathcal{I}\otimes S_i(|\Psi\rangle\langle\Psi|)) = \sum_i \frac{\mathrm{tr}\mathscr{T}(S_i)}{d_A d_B}\log\frac{||\mathscr{T}(S_i^\Gamma)||_1}{\mathrm{tr}\mathscr{T}(S_i)}, \tag{71}$$

Note for the upper bounds the denominator p_i could be removed due to concavity (see (53)), which can't be applied here. Also note by Corollary 1 and (69) there are several expressions for these bounds, just like sub-operations.

4.4 Proposition 2

As defined in the main paper, for two operators or linear mappings X and Y

$$D_{p,\Gamma}(X,Y) := ||X-Y||_{p,\Gamma} := ||X^\Gamma - Y^\Gamma||_p, \tag{72}$$

where

$$||L||_p := ||\mathscr{T}(L)||_p \text{ for any linear mappings } L. \tag{73}$$

As discussed in Sec. 1.5, partial transpositions on both operators and linear mappings are isomorphisms.

Now let's prove:

Proposition 2. *For any density operators ρ and ρ_i, and deterministic operations S and S_i, we have an equality*

$$(S_2^\Gamma - S_1^\Gamma)^\dagger_+(I) = (S_2^\Gamma - S_1^\Gamma)^\dagger_-(I), \tag{74}$$

and the following inequalities:

$$D_{1,\Gamma}\left(S_1(\rho), S_2(\rho)\right) \le 2||(S_2^\Gamma - S_1^\Gamma)^\dagger_\pm(I)||\,||\rho^\Gamma||_1 \tag{75}$$

$$\le D_{1,\Gamma}(S_1,S_2)||\rho^\Gamma||_1, \tag{76}$$

$$D_{1,\Gamma}\left(S(\rho_1), S(\rho_2)\right) \le \left(1 + 2||{S_-^\Gamma}^\dagger(I)||\right) D_{1,\Gamma}(\rho_1,\rho_2). \tag{77}$$

Proof. Because S_i are deterministic, ${S_i^\Gamma}^\dagger(I) = I$, and

$$(S_2^\Gamma - S_1^\Gamma)^\dagger(I) = 0.$$

Hence we can phrase our problem this way: If an HP mapping L has $L^\dagger(I)=0$, given an operator O with a fixed trace norm, how large can $||L(O)||_1$ be? Let's begin with

$$L^\dagger(I) = L^\dagger_+(I) - L^\dagger_-(I) = 0 \Rightarrow L^\dagger_+(I) = L^\dagger_-(I), \tag{78}$$

confirming $(S_2^\Gamma - S_1^\Gamma)^\dagger_+(I) = (S_2^\Gamma - S_1^\Gamma)^\dagger_-(I)$. Then by (46),

$$\begin{aligned}||L(O)||_1 &= \mathrm{tr}L(O)^+ + \mathrm{tr}L(O)^- \\ &\le \mathrm{tr}(L_+ + L_-)(O^+ + O^-) \\ &= 2\mathrm{tr}\left[L^\dagger_\pm(I)(O^+ + O^-)\right] \\ &\le 2||L^\dagger_\pm(I)||\,||O||_1 \text{ by (14) and } ||O||_1 = \mathrm{tr}(O^+ + O^-).\end{aligned}$$

(75) is proven. To show (76), we need the inequality $||O|| \le ||O||_1$ [19, 27], so $||(S_2^\Gamma - S_1^\Gamma)^\dagger_\pm(I)|| \le ||(S_2^\Gamma - S_1^\Gamma)^\dagger_\pm(I)||_1$. By Corollary 1, $\mathrm{tr}(S_2^\Gamma - S_1^\Gamma)^\dagger_\pm(I) = \mathrm{tr}\mathscr{T}(S_2^\Gamma - S_1^\Gamma)^\pm$, so by (78)

$$2\mathrm{tr}\mathscr{T}(S_2^\Gamma - S_1^\Gamma)^\pm = \mathrm{tr}\mathscr{T}(S_2^\Gamma - S_1^\Gamma)^+ + \mathrm{tr}\mathscr{T}(S_2^\Gamma - S_1^\Gamma)^- = ||\mathscr{T}(S_2^\Gamma - S_1^\Gamma)||_1 = ||S_2 - S_1||_{1,\Gamma}.$$

To show (77) is pretty similar, as

$$S(\rho_1)^\Gamma - S(\rho_2)^\Gamma = S^\Gamma[(\rho_1-\rho_2)^\Gamma].$$

Setting $O=(\rho_1-\rho_2)^\Gamma$, by (46) we can easily find

$$\begin{aligned}||S^\Gamma(O)||_1 &\le \mathrm{tr}(S_+^\Gamma+S_-^\Gamma)(O^+ + O^-)\\ &\le ||S_+^{\Gamma\dagger}(I)+S_-^{\Gamma\dagger}(I)||\ ||O||_1\\ &= \left(1+2||S_-^{\Gamma\dagger}(I)||\right)||\rho_1-\rho_2||_{1,\Gamma} \text{ by } (43).\end{aligned}$$

□

4.5 Upper bounds with an ancilla

Since $\widetilde{L}_\pm$ are CP, so are $\mathcal{I}\otimes\widetilde{L}_\pm$, and $\mathcal{I}\otimes(\widetilde{L}_+-\widetilde{L}_-)=\mathcal{I}\otimes\widetilde{L}_+-\mathcal{I}\otimes\widetilde{L}_-$, which is therefore a decomposition of $\mathcal{I}\otimes L$ as a difference of CP mappings, or in our notation, $\widetilde{\mathcal{I}\otimes L}_\pm$.

With this in mind, let's show the upper bounds are valid with an ancilla; in particular [6]

$$||(\mathcal{I}\otimes\widetilde{S_i^\Gamma}_-)^\dagger(I)|| = ||\widetilde{S_i^\Gamma}_-^\dagger(I)||, \tag{79}$$

where $\mathcal{I}$ operates on the ancilla. Namely, the bounds obtained for S_i are also applicable to $\mathcal{I}\otimes S_i$ and vice versa. Note $(\mathcal{I}\otimes S_i)^\Gamma=\mathcal{I}\otimes S_i^\Gamma$. Unlike in Sec. 4.3, the ancilla can be of any dimension, not merely a clone of the original system.

Here let's prove another corollary of Lemma 3:

Corollary 2. *For any linear mapping L,*

$$(\mathcal{I}\otimes L)^\dagger(I) = I\otimes L^\dagger(I).$$

If this is true, then $||(\mathcal{I}\otimes L)^\dagger(I)||=||I||\,||L^\dagger(I)||=||L^\dagger(I)||$, whereby we can show (79).

Proof. Choose $\{|e_i\rangle\}$ as the orthonormal basis of the ancilla, and define $G_{ij}:=|e_i\rangle\langle e_j|$. We can find

$$\mathscr{T}(\mathcal{I}\otimes L)=\Big(\sum_{i,j}G_{ij}\otimes G_{ij}\Big)\otimes\mathscr{T}(L).$$

By Lemma 3, $(\mathcal{I}\otimes L)^\dagger(I)=\mathrm{tr}_2\mathscr{T}(\mathcal{I}\otimes L)^*$. Because $G_{ij}^*=G_{ij}$,

$$\begin{aligned}\mathrm{tr}_2\mathscr{T}(\mathcal{I}\otimes L)^* &= \mathrm{tr}_2\Big(\sum_{i,j}G_{ij}\otimes G_{ij}\Big)\otimes\mathrm{tr}_2\mathscr{T}(L)^*\\ &=\sum_{i,j}G_{ij}\delta_{ij}\otimes L^\dagger(I)\\ &= I\otimes L^\dagger(I)\end{aligned}$$

The reader can find a discussion on complex conjugation of operators in Sec. 3.2. This proof can be compared with that in Ref. [6]. □

4.6 Upper bounds using Schatten p-norm

All the upper bounds come from

$$\mathrm{tr}P(H^+ + H^-),$$

where P originates from the adjoint of a CP mapping. By matrix Hölder inequality (16) [20],

$$\mathrm{tr}P(H^+ + H^-)\le ||P||_p||H^+ + H^-||_q, \tag{80}$$

where $1\le p,q\le\infty$ and $1/p+1/q=1$. We do not need to take the absolute value as $\mathrm{tr}P_1P_2\ge 0$.

For a Hermitian operator H,

$$|H| = H^+ + H^-,$$

because H^+ and H^- have orthogonal eigenvectors, c.f. Lemma 1. Therefore,

$$||H^+ + H^-||_p = ||H||_p,$$

and (80) becomes

$$\mathrm{tr}P(H^+ + H^-) \le ||P||_p||H||_q. \tag{81}$$

Using (81), we can generalize the upper bounds we've obtained. For example, the upper bound for negativity from part 1 of Proposition 1 becomes

$$EC_N(S) \le ||\widetilde{S^{\Gamma}}^{\dagger}_{-}(I)||_p||\rho^{\Gamma}||_q,$$

and the upper bound for logarithmic negativity will be state-dependent when $q \neq 1$. Or for (77), we may want to use another norm for states, then the norm for $S^{\Gamma}_{-}(I)$ can be adjusted accordingly. The rest can be derived easily.

5 Equivalence of the bounds

By part 2 of Proposition 1, $\mathrm{EC}_L(S) \le \log(1 + 2||\sum_i S^{\Gamma}_i{}^{\dagger}_{-}(I)||)$; be aware that here it is $S^{\Gamma}_{i}{}_{-}$ instead of $\widetilde{S^{\Gamma}_{i}}_{-}$. In Ref. [6], it was shown for a deterministic operation $S = \sum_i S_i$, where $S_i(\rho) = V_i\rho V_i^{\dagger}$ and V_i has a Schmidt decomposition [28]

$$V_i = \sum_{j(i)} \lambda_{ij} A_{ij} \otimes B_{ij}, \tag{82}$$

the entangling capacity has an upper bound:

$$\mathrm{EC}_L(S) \le \log\big(\sum_i || \sum_{j(i)} \lambda_{ij} A^{\dagger}_{ij} A_{ij}|| \, || \sum_{k(i)} \lambda_{ik} B^{\dagger}_{ik} B_{ik}||\big).$$

It was shown by Campbell for a unitary operator $U = \sum_i \lambda_i A_i \otimes B_i$ the lower bound is [6]

$$\log \frac{(\sum_i \lambda_i)^2}{d_A d_B}, \tag{83}$$

while our lower bound is

$$\log\left(1 + 2\frac{||S^{\Gamma}_{-}{}^{\dagger}(I)||_1}{d_A d_B}\right). \tag{84}$$

Here we would like to show

Lemma 5. *For deterministic $S = \sum_i S_i$, where $S_i = V_i\rho V_i^{\dagger}$ and the Schmidt decompositions of V_i are $V_i = \sum_{j(i)} \lambda_{ij} A_{ij} \otimes B_{ij}$,*

$$\begin{aligned} 1 + 2||\sum_i S^{\Gamma}_i{}^{\dagger}_{-}(I)|| &= ||\sum_i \sum_{j(i)} \lambda_{ij} A^{*}_{ij}{}^{\dagger} A^{*}_{ij} \otimes \sum_{k(i)} \lambda_{ik} B^{\dagger}_{ik} B_{ik}|| \\ &\le \sum_i || \sum_{j(i)} \lambda_{ij} A^{\dagger}_{ij} A_{ij}|| \, || \sum_{k(i)} \lambda_{ik} B^{\dagger}_{ik} B_{ik}||, \end{aligned}$$

For a unitary operation $S(\rho) = U\rho U^{\dagger}$, where the Schmidt decomposition of U is $U = \sum_i \lambda_i A_i \otimes B_i$,

$$1 + 2\frac{||S^{\Gamma}_{-}{}^{\dagger}(I)||_1}{d_A d_B} = \frac{(\sum_i \lambda_i)^2}{d_A d_B}.$$

By which, $\mathrm{EC}_L(S) \le \log(1 + 2||\sum_i S^{\Gamma}_i{}^{\dagger}_{-}(I)||) \le \big(\sum_i || \sum_{j(i)} \lambda_{ij} A^{\dagger}_{ij} A_{ij}|| \, || \sum_{k(i)} \lambda_{ik} B^{\dagger}_{ik} B_{ik}||\big)$, and the lower bound obtained in Ref. [6], (83), is the same as that from Proposition 1, (84).

5.1 Upper bound

Utilizing the tricks from Appendix E of Ref. [6], we are able to give the following proof.

5.1.1 Single sub-operation

Let's first consider just one sub-operation S_i, and drop the subscript i for this moment. Suppose $S(O) = VOV^\dagger$, with $V = \sum_i \lambda_i A_i \otimes B_i$ being the Schmidt decomposition. Then

$$S^\Gamma(O) = (VO^\Gamma V^\dagger)^\Gamma = \sum_{i,j} \lambda_i \lambda_j A_j^* \otimes B_i O (A_i^* \otimes B_j)^\dagger. \tag{85}$$

Define

$$\begin{aligned} V_{ij}^\pm &:= \left(A_i^* \otimes B_j \pm A_j^* \otimes B_i \text{ for } \right)/\sqrt{2} \text{ for } i \neq j, \\ V_{ii}^+ &:= A_i^* \otimes B_i. \end{aligned} \tag{86}$$

We can see for $i \neq j$

$$A_j^* \otimes B_i O (A_i^* \otimes B_j)^\dagger + A_i^* \otimes B_j O (A_j^* \otimes B_i)^\dagger = V_{ij}^+ O V_{ij}^{+\dagger} - V_{ij}^- O V_{ij}^{-\dagger}; \tag{87}$$

Note $V_{ij}^\pm = \pm V_{ji}^\pm$ and therefore $V_{ij}^- O V_{ij}^- = V_{ji}^- O V_{ji}^-$. We now have

$$S^\Gamma(O) = \sum_i \lambda_i^2 V_{ii}^+ O V_{ii}^{+\dagger} + \frac{1}{2} \sum_{i \neq j} \lambda_i \lambda_j V_{ij}^+ O V_{ij}^{+\dagger} - \frac{1}{2} \sum_{i \neq j} \lambda_i \lambda_j V_{ij}^- O V_{ij}^{-\dagger}. \tag{88}$$

As each summation on the right side of (88) is in the Kraus form, each is a CP mapping, so we have

$$\begin{aligned} \widetilde{S^\Gamma}_+(O) &= \sum_i \lambda_i^2 V_{ii}^+ O V_{ii}^{+\dagger} + \frac{1}{2} \sum_{i \neq j} \lambda_i \lambda_j V_{ij}^+ O V_{ij}^{+\dagger} \\ \widetilde{S^\Gamma}_-(O) &= \frac{1}{2} \sum_{i \neq j} \lambda_i \lambda_j V_{ij}^- O V_{ij}^{-\dagger}. \end{aligned} \tag{89}$$

Now the problems is, is $\widetilde{S^\Gamma}_\pm = S^\Gamma_\pm$? This is equivalent to $\mathscr{T}(\widetilde{S^\Gamma}_\pm)$ having orthogonal eigenvectors, by Lemma 1 (and Choi isomorphism, well, being bijective). We can find $\{V_{ij}^+\}$ and $\{V_{kl}^-\}$ are mutually orthogonal:

$$(V_{ij}^+ | V_{ij}^-) = \left(A_i^* \otimes B_j + A_j^* \otimes B_j | A_i^* \otimes B_j - A_j^* \otimes B_i\right)/2 = 0; \tag{90}$$

the orthogonality of the rest can be shown by the orthonormality of $\{A_i\}$ and $\{B_i\}$. Choose an orthonormal basis $\{|a_i\rangle\}$ for the composite system $A \otimes B$ whose dimension is $d = d_A d_B$. Like (56), define

$$|\Psi\rangle := \frac{1}{\sqrt{d}} \sum_i |a_i\rangle |a_i\rangle. \tag{91}$$

Then the Choi isomorphism of an operation by a single $V_{kl}^\pm$ is

$$\sum_{ij} |a_i\rangle\langle a_j| \otimes V_{kl}^\pm |a_i\rangle\langle a_j| V_{kl}^{\pm\dagger} = d(I \otimes V_{kl}^\pm)|\Psi\rangle\langle\Psi|(I \otimes V_{kl}^\pm)^\dagger; \tag{92}$$

Therefore $\mathscr{T}(\widetilde{S^\Gamma}_\pm)$ are ensembles of pure states. It was proved in Appendix F of Ref. [6] that if operators O_1 and O_2 are orthogonal, then $I \otimes O_1 |\Psi\rangle$ and $I \otimes O_2 |\Psi\rangle$ are orthogonal:

$$\langle\Psi| (I \otimes O_2)^\dagger I \otimes O_1 |\Psi\rangle = \frac{1}{d} \sum_{i,j} \langle a_i | a_j\rangle \langle a_i | O_2^\dagger O_1 | a_j\rangle = \frac{1}{d}(O_2 | O_1). \tag{93}$$

By Lemma 2, $\mathscr{T}(\widetilde{S^\Gamma}_\pm)$ have orthogonal eigenvectors, so $\widetilde{S^\Gamma}_\pm$ as defined in (89) are equal to $S^\Gamma_\pm$. Therefore,

$$\begin{aligned} S_-^{\Gamma\dagger}(I) &= \frac{1}{2} \sum_{i \neq j} \lambda_i \lambda_j V_{ij}^{-\dagger} V_{ij}^-, \\ S_+^{\Gamma\dagger}(I) &= \sum_i \lambda_i^2 V_{ii}^{+\dagger} V_{ii}^+ + \frac{1}{2} \sum_{i \neq j} \lambda_i \lambda_j V_{ij}^{+\dagger} V_{ij}^+, \end{aligned}$$

and

$$S_+^{\Gamma\dagger}(I) + S_-^{\Gamma\dagger}(I) = \sum_i \lambda_i^2 A_i^{*\dagger} A_i^* \otimes B_i^\dagger B_i + \frac{1}{2}\sum_{i\neq j} \lambda_i\lambda_j \left(A_i^{*\dagger} A_i^* \otimes B_j^\dagger B_j + A_j^{*\dagger} A_j^* \otimes B_i^\dagger B_i\right)$$
$$= \sum_{i=j} \lambda_i\lambda_j A_i^{*\dagger} A_i^* \otimes B_j^\dagger B_j + \sum_{i\neq j} \lambda_i\lambda_j A_i^{*\dagger} A_i^* \otimes B_j^\dagger B_j$$
$$= \sum_i \lambda_i A_i^{*\dagger} A_i^* \otimes \sum_j \lambda_j B_j^\dagger B_j. \tag{94}$$

5.1.2 In the entirety

Let's come back to the operation $S = \sum_i S_i$, where $S_i(A) = V_i A V_i^\dagger$ and $V_i = \sum_j \lambda_j A_{ij} \otimes B_{ij}$ is the Schmidt decomposition. By (43), for a TP $L = \sum_i L_i$ with each L_i being HP, we have

$$1 + 2\Big|\Big|\sum_i {L_i}_-^\dagger(I)\Big|\Big| = \Big|\Big|\sum_i \left({L_i}_+^\dagger(I) + {L_i}_-^\dagger(I)\right)\Big|\Big|; \tag{95}$$

along with (94) we obtain,

$$1 + 2||\sum_i {S_i^\Gamma}_-^\dagger(I)|| = ||\sum_i \left({S_i^\Gamma}_+^\dagger(I) + {S_i^\Gamma}_-^\dagger(I)\right)||$$
$$= ||\sum_i \sum_{j(i)} \lambda_{ij} {A_{ij}^*}^\dagger A_{ij}^* \otimes \sum_{k(i)} \lambda_{ik} B_{ik}^\dagger B_{ik}||$$
$$\leq \sum_i ||\sum_{j(i)} \lambda_{ij} {A_{ij}^*}^\dagger A_{ij}^*||\,||\sum_{k(i)} \lambda_{ik} B_{ik}^\dagger B_{ik}||$$
$$= \sum_i ||\sum_{j(i)} \lambda_{ij} {A_{ij}}^\dagger A_{ij}||\,||\sum_{k(i)} \lambda_{ik} B_{ik}^\dagger B_{ik}||.$$

5.2 Lower bound

By (44),

$$1 + 2\frac{||S_-^{\Gamma\dagger}(I)||_1}{d_A d_B} = \frac{||S_+^{\Gamma\dagger}(I) + S_-^{\Gamma\dagger}(I)||_1}{d_A d_B} \tag{96}$$

and by (94),

$$||S_+^{\Gamma\dagger}(I) + S_-^{\Gamma\dagger}(I)||_1 = \sum_i \lambda_i \mathrm{tr} A_i^{*\dagger} A_i^* \sum_j \lambda_j \mathrm{tr} \lambda_j B_j^\dagger B_j = (\sum_i \lambda_i)^2 \tag{97}$$

because $\{A_i\}$ and $\{B_i\}$ are orthonormal sets of operators. (96) and (97) together demonstrate the equivalence.

6 PPT and separable unitary operations

Proposition 3. *In a finite-dimensional system, the following statements for either a unitary operation $S(\rho) = U\rho U^\dagger$ or a pure state $|\psi\rangle$ are equivalent:*

1. *The Schmidt rank of U or $|\psi\rangle$ is 1.*
2. *The operation/state is separable.*
3. *The operation/state is PPT.*

That PPT pure states are necessarily separable has already been shown in, e.g. [21, 29], where the negativity of a pure state $|\psi\rangle$ was explicitly calculated, given its Schmidt decomposition. Below we will provide an alternative proof for pure states.

6.1 Proof for pure states

By definition a pure state is separable if and only if its Schmidt rank is 1. Suppose the Schmidt decomposition of a pure state is

$$|\psi\rangle = \sum_i \lambda_i |a_i\rangle|b_i\rangle. \tag{98}$$

Since partial transpose with respect to different bases are related unitarily, we can do this with respect to any basis, and let's choose $\{|a_i\rangle\}$. We obtain

$$\rho^\Gamma = (|\psi\rangle\langle\psi|)^\Gamma = \sum_{i,j} \lambda_i\lambda_j |a_j\rangle\langle a_i| \otimes |b_i\rangle\langle b_j|. \tag{99}$$

There's a decomposition of ρ^Γ as $\rho^\Gamma = \widetilde{\rho^\Gamma}^+ - \widetilde{\rho^\Gamma}^-$, where

$$\widetilde{\rho^\Gamma}^+ = \sum_i \lambda_i^2 (|a_i\rangle|b_i\rangle)(\langle a_i|\langle b_i|) + \sum_{i<j} \lambda_i\lambda_j \frac{|a_i\rangle|b_j\rangle + |a_j\rangle|b_i\rangle}{\sqrt{2}} \frac{\langle a_i|\langle b_j| + \langle a_j|\langle b_i|}{\sqrt{2}}, \tag{100a}$$

$$\widetilde{\rho^\Gamma}^- = \sum_{i<j} \lambda_i\lambda_j \frac{|a_i\rangle|b_j\rangle - |a_j\rangle|b_i\rangle}{\sqrt{2}} \frac{\langle a_i|\langle b_j| - \langle a_j|\langle b_i|}{\sqrt{2}}. \tag{100b}$$

As the vectors are orthonormal, (100a) and (100a) together are eigendecomposition of ρ^Γ; in other words $\widetilde{\rho^\Gamma}^\pm = \rho^{\Gamma\pm}$. Therefore, $\rho^{\Gamma-} = 0$, i.e. ρ is PPT if and only if the Schmidt rank of $|\psi\rangle$ is 1.

6.2 Proof for unitary operations

First, it's easy to see a unitary operation $S(O) = UOU^\dagger$ is separable if U has Schmidt rank 1. To show it's separable only if the Schmidt rank is 1, with $U = \sum_i \lambda_i A_i \otimes B_i$ and (91):

$$\begin{aligned}
I_{AB} \otimes U|\Psi_{AB}\rangle &= \frac{1}{\sqrt{d_A d_B}} \sum_{i,j,k} \lambda_i I_{AB} \otimes (A_i \otimes B_i)|a_j\rangle|b_k\rangle|a_j\rangle|b_k\rangle \\
&= \frac{1}{\sqrt{d_A d_B}} \sum_i \lambda_i \Big(I_A \otimes A_i \sum_j |a_j\rangle|a_j\rangle\Big) \otimes \Big(\sum_k I_B \otimes B_i |a_k\rangle|a_k\rangle\Big) \\
&= \sum_i \lambda_i (I_A \otimes A_i|\Psi_A\rangle) \otimes (I_B \otimes B_i|\Psi_B\rangle).
\end{aligned} \tag{101}$$

If U is separable, then the state above is separable with respect to A and B. Because of (93), $I_A \otimes A_i|\Psi_A\rangle$ are orthogonal with $I_A \otimes A_j|\Psi_A\rangle$ for $i \neq j$, likewise for B. Therefore, (101) is a Schmidt decomposition of $I_{AB} \otimes U|\Psi_{AB}\rangle$ (after normalizing $I_A \otimes A_i|\Psi_A\rangle$ and $I_B \otimes B_i|\Psi_B\rangle$), and this vector is separable if and only if the Schmidt rank of U is 1. Therefore, no unitary operators with Schmidt rank higher than 1 are separable, so a unitary operator is separable if and only if its Schmidt rank is 1.

From (86) and (89), it's clear that a unitary operation is PPT if and only if there exist no V_{ij}^-, which happens if and only if the Schmidt rank of the unitary operator is 1. Thus a unitary operation is PPT if and only if it's separable.

(96) and (97) also confirm this: The unitary operation is PPT if and only if $\mathrm{tr}\mathscr{T}(S^\Gamma)^- = ||S_-^{\Gamma\dagger}(I)||_1 = 0$, i.e. $(\sum_i \lambda_i)^2 = d_A d_B$. Because each $\lambda_i > 0$, $(\sum_i \lambda_i)^2 \geq \sum_i \lambda_i^2$, and they are equal if and only if there's only one λ_i. Since

$$\mathrm{tr}U^\dagger U = \mathrm{tr}I = (U|U) = (\sum_i \lambda_i A_i \otimes B_i | \sum_j \lambda_j A_j \otimes B_j) = d_A d_B, \tag{102}$$

for $(\sum_i \lambda_i)^2$ to become $d_A d_B$ the Schmidt rank of U must be 1, i.e. separable. □

7 Schmidt decomposition and eigendecomposition

The method from Ref. [6] used to show Lemma 5 and Proposition 3 can be further generalized as follows.

As was shown in Sec. 5.1, for a (sub-)operation $S(A) = VAV^\dagger$ where the Schmidt decomposition of V is $\sum_i \lambda_i A_i \otimes B_i$, $S_\pm^\Gamma$ can be determined from the Schmidt decomposition of V, in particular (86). Let's express

(89) as

$$S_+^\Gamma(O) := \sum_i \lambda_i^2 V_{ii}^+ O V_{ii}^{+\dagger} + \sum_{i<j} \lambda_i \lambda_j V_{ij}^+ O V_{ij}^{+\dagger}$$
$$S_-^\Gamma(O) := \sum_{i<j} \lambda_i \lambda_j V_{ij}^- O V_{ij}^{-\dagger}. \tag{103}$$

Because both $\{A_i\}$ and $\{B_i\}$ are composed of orthonormal operators, $(V_{ij}^\pm|V_{kl}^\pm) = \delta_{ik}\delta_{jl}$, and thus $\{V_{ij}^+ : i \le j\}$ and $\{V_{ij}^- : i < j\}$ are sets of orthonormal operators, and $V_{ij}^+ \perp V_{kl}^-$. Therefore after Choi isomorphism, $\mathscr{T}(S^\Gamma)$ becomes an ensemble of orthogonal pure states, c.f. (92) and (93), giving us an eigendecomposition of it.

To find the eigenvalues and eigenvectors of $\mathscr{T}(S^\Gamma)$, because $\{V_{ij}^+ : i \le j\}$ and $\{V_{ij}^- : i < j\}$ are normalized and because of (93), we can easily see $\mathscr{T}(S^\Gamma)$ has

$$\text{eigenvectors } \sqrt{d} I \otimes V_{ij}^\pm |\Psi\rangle \text{ with corresponding eigenvalues} \pm \lambda_i\lambda_j,\ i \le j \text{ for } V_{ij}^+ \text{ and } i<j \text{ for } V_{ij}^-. \tag{104}$$

For example, for a unitary operation $S(A) = UAU^\dagger$ where $U = \sum_{i=1}^2 \lambda_i A_i \otimes B_i$ with Schmidt rank 2, $\mathscr{T}(S^\Gamma)$ has three positive eigenvalues and a negative one: λ_1^2, λ_2^2, and $\pm\lambda_1\lambda_2$.

In general, given a linear mapping L of this form (c.f. (85)):

$$L(O) = \sum_{i,j} A_i \otimes B_j O (A_j \otimes B_i)^\dagger, \tag{105}$$

we can find a decomposition of L: $L = \widetilde{L}_+ - \widetilde{L}_-$. If $\{A_i\}$ and $\{B_i\}$ are sets of orthogonal operators, then $\widetilde{L}_\pm = L_\pm$, and we can determine the spectrum of $\mathscr{T}(L)$ by $\{A_i\}$ and $\{B_i\}$ alone.

Similarly, with an operator of the form

$$H = \sum_{i,j} |v_j\rangle\langle v_i| \otimes |w_i\rangle\langle w_j|, \tag{106}$$

where $|v_i\rangle$ and $|w_i\rangle$ aren't necessarily orthonormal, we can obtain

$$\widetilde{H}^+ = \sum_i (|v_i\rangle|w_i\rangle)(\langle v_i|\langle w_i|) + \frac{1}{2}\sum_{i<j}(|v_i\rangle|w_j\rangle + |v_j\rangle|w_i\rangle)(\langle v_i|\langle w_j| + \langle v_j|\langle w_i|)$$
$$\widetilde{H}^- = \frac{1}{2}\sum_{i<j}(|v_i\rangle|w_j\rangle - |v_j\rangle|w_i\rangle)(\langle v_i|\langle w_j| - \langle v_j|\langle w_i|). \tag{107}$$

If $\{|v_i\rangle\}$ and $\{|v_i\rangle\}$ are orthogonal sets of vectors, this gives us the eigendecomposition of H.

8 Norms

8.1 Upper bound of $||S_-^{\Gamma\dagger}(I)||$

To prove the inequality

$$||S_-^{\Gamma\dagger}(I)|| \le ||S_-^{\Gamma\dagger}(I)||_1 = (||S||_{1,\Gamma} - d_A d_B)/2$$

is quite easy: By the inequality $||O|| \le ||O||_1$ [19, 27], $||S_-^{\Gamma\dagger}(I)|| \le ||S_-^{\Gamma\dagger}(I)||_1 = \mathrm{tr} S_-^{\Gamma\dagger}(I)$; by Corollary 1, $\mathrm{tr} S_-^{\Gamma\dagger}(I) = \mathrm{tr}\mathscr{T}(S_-^\Gamma) = ||\mathscr{T}(S_-^\Gamma)||_1$. By (42) and (45), $||\mathscr{T}(S^\Gamma)||_1 = \mathrm{tr}\mathscr{T}(S^\Gamma) + 2\mathrm{tr}\mathscr{T}(S_-^\Gamma) = d_A d_B + 2\mathrm{tr}\mathscr{T}(S_-^\Gamma)$, so $2\mathrm{tr}S_-^{\Gamma\dagger}(I) = ||S||_{1,\Gamma} - d_A d_B$.

The difference between the two sides of the inequality, $||S_-^{\Gamma\dagger}(I)||_1 - ||S_-^{\Gamma\dagger}(I)|| = \mathrm{tr}\mathscr{T}(S_-^\Gamma) - ||S_-^{\Gamma\dagger}(I)||$, is the sum of the eigenvalues of $S_-^{\Gamma\dagger}(I)$ minus the largest one. When the operation is PPT, the right side of the inequality is zero.

8.2 Equivalence of norms

Here we will show

$$\frac{||H||_1}{\min(d_A, d_B)} \le ||H||_{1,\Gamma} \le \min(d_A, d_B)||H||_1. \tag{108}$$

By Ref. [21], for a state with Schmidt decomposition $|\psi\rangle = \sum_i \lambda_i |a_i\rangle|b_i\rangle$

$$||(|\psi\rangle\langle\psi|)^\Gamma||_1 = ||(|\psi\rangle\langle\psi|)||_{1,\Gamma} = \Big(\sum_i \lambda_i\Big)^2. \tag{109}$$

This can be proved with the aid of a unitary operator as in Ref. [21], or

$$[(|\psi\rangle\langle\psi|)^\Gamma]^2 = \sum_i \lambda_i^2 |a_i\rangle\langle a_i| \otimes \sum_j \lambda_j^2 |b_j\rangle\langle b_j|, \tag{110}$$

so $|(|\psi\rangle\langle\psi|)^\Gamma| = \sum_{i,j} \lambda_i\lambda_j |a_i\rangle\langle a_i| \otimes |b_j\rangle\langle b_j|$ and $||(|\psi\rangle\langle\psi|)^\Gamma||_1 = (\sum_i \lambda_i)^2$.

Because the Schmidt rank is at most $\min(d_A, d_B)$, by Schwarz inequality

$$\Big(\sum_i \lambda_i\Big)^2 \le \sum_i \lambda^2 \sum_j 1^2 = \min(d_A, d_B). \tag{111}$$

Hence, for any Hermitian operator H with spectral decomposition $H = \sum_i h_i |\psi_i\rangle\langle\psi_i|$

$$||H^\Gamma||_1 = ||H||_{1,\Gamma} \le \sum_i |h_i|\, ||(|\psi_i\rangle\langle\psi_i|)||_{1,\Gamma} \le \min(d_A, d_B) \sum_i |h_i| = \min(d_A, d_B)||H||_1. \tag{112}$$

Since $(H^\Gamma)^\Gamma = H$ (and because Γ is HP), for the same reason $||H||_1 \le \min(d_A, d_B)||H^\Gamma||_1 = \min(d_A, d_B)||H||_{1,\Gamma}$.

As for HP mappings, $\mathscr{T}(L) \in \mathcal{L}(\mathcal{H}_{A_1} \otimes \mathcal{H}_{A_2} \otimes \mathcal{H}_{B_1} \otimes \mathcal{H}_{B_2})$, and $\Gamma = \mathcal{T}_{A_1A_2} \otimes \mathcal{I}_{B_1B_2}$. The Schmidt rank of a bipartite state, with the parties being $(\mathcal{H}_{A_1} \otimes \mathcal{H}_{A_2})$ and $(\mathcal{H}_{B_1} \otimes \mathcal{H}_{B_2})$, is at most $\min(d_{A_1}d_{A_2}, d_{B_1}d_{B_2})$, so

$$\frac{||L||_1}{\min(d_{A_1}d_{A_2}, d_{B_1}d_{B_2})} \le ||L||_{1,\Gamma} \le \min(d_{A_1}d_{A_2}, d_{B_1}d_{B_2})||L||_1. \tag{113}$$

For non-Hermitian operators and non-HP mappings, we except similar relations to exist.

9 Reaching the entangling capacity

9.1 Proposition 4

Let's show the following proposition:

Proposition 4. *Suppose $\widetilde{S^\Gamma}_\pm = S^\Gamma_\pm$. Let* ran *denote the range or image of a mapping, and*

$$S^\Gamma_\pm(O) = \sum_i c_i^\pm V_i^\pm O V_i^{\pm\dagger},\ c_i^\pm > 0, \tag{114}$$

be operator-sum representations of $S^\Gamma_\pm$, and

$$\rho^{\Gamma\pm} = \sum_i |\psi_i^\pm\rangle\langle\psi_i^\pm| \tag{115}$$

be ensembles of $\rho^{\Gamma\pm}$. The upper bound of the entangling capacity given by Proposition 1 is reached if and only if the two conditions below are both satisfied:

1. ran $\Big[S^\Gamma_-(\rho^{\Gamma+}) + S^\Gamma_+(\rho^{\Gamma-})\Big]$ *and* ran $\Big[S^\Gamma_+(\rho^{\Gamma+}) + S^\Gamma_-(\rho^{\Gamma-})\Big]$ *are orthogonal, which is equivalent to the orthogonality of the following vectors:*

$$\langle V_i^\pm \psi_j^\mp | V_k^+ \psi_l^+\rangle = 0 \text{ and } \langle V_i^\pm \psi_j^\mp | V_k^- \psi_l^-\rangle = 0\ \forall i,j,k,l. \tag{116}$$

2. ranρ^Γ *is a subspace of the eigenspace relative to the largest eigenvalue of ${S^\Gamma_\pm}^\dagger(I)$.*

The second condition above is satisfied by any state ρ when ${S^\Gamma_\pm}^\dagger(I) \propto I$. Besides, the upper and lower bounds in (41) are the same if and only if ${S^\Gamma_\pm}^\dagger(I) \propto I$.

Proof. First, for $P \geq 0$, $||P||_1/d = \mathrm{tr}P/d$, where d is the dimension of the space, is the average of its eigenvalues (zero included), whereas $||P||$ is its largest eigenvalue. Hence they're the same if and only if $P \propto I$, and $||S_{\pm}^{\Gamma\dagger}(I)||_1/(d_A d_B) = ||S_{\pm}^{\Gamma\dagger}(I)||$ if and only if $S_{\pm}^{\Gamma\dagger}(I) \propto I$.

For the rest of the proof please read the proof of Lemma 4 if necessary. For $\mathrm{tr}[S_{-}^{\Gamma\dagger}(I)(\rho^{\Gamma+} + \rho^{\Gamma-})]$ to equal $||S_{-}^{\Gamma\dagger}(I)||\,||\rho^{\Gamma}||_1$, it's necessary and sufficient that V is a subspace of the eigenspace of $S_{-}^{\Gamma\dagger}(I)$'s largest eigenvalue, i.e. the second condition, and it's satisfied when $S_{-}^{\Gamma\dagger}(I) \propto I$; see the discussion below (14). Note because $S_{+}^{\Gamma\dagger}(I) = S_{-}^{\Gamma\dagger}(I) + I$, their spectra differ by 1 exactly.

For $\mathrm{tr}(S(\rho)^{\Gamma})^{-} = \mathrm{tr}(S^{\Gamma}(\rho^{\Gamma}))^{-}$ and $\mathrm{tr}S_{+}^{\Gamma}(\rho^{\Gamma-}) + \mathrm{tr}S_{-}^{\Gamma}(\rho^{\Gamma+})$ to be equal, by Lemma 1 it's necessary and sufficient that the eigenvectors of $S_{-}^{\Gamma}(\rho^{\Gamma+}) + S_{+}^{\Gamma}(\rho^{\Gamma-})$ and those of $S_{+}^{\Gamma}(\rho^{\Gamma+}) + S_{-}^{\Gamma}(\rho^{\Gamma-})$ are orthogonal with each other, that is, they are the eigendecomposition $S^{\Gamma}(\rho^{\Gamma})$. From (114) and (115) we acquire ensembles of $S_{\pm}^{\Gamma}(\rho^{\Gamma+}) + S_{\mp}^{\Gamma}(\rho^{\Gamma-})$:

$$S_{\pm}^{\Gamma}(\rho^{\Gamma+}) + S_{\mp}^{\Gamma}(\rho^{\Gamma-}) = \sum_{i,j} c_i^{\pm} V_i^{\pm} |\tilde{\psi}_j^{+}\rangle\langle\tilde{\psi}_j^{+}| V_i^{\pm\dagger} + \sum_{k,l} c_k^{\mp} V_k^{\mp} |\tilde{\psi}_l^{-}\rangle\langle\tilde{\psi}_l^{-}| V_k^{\mp\dagger}. \tag{117}$$

By Lemma 2, the eigenvectors of $S_{-}^{\Gamma}(\rho^{\Gamma+}) + S_{+}^{\Gamma}(\rho^{\Gamma-})$ and those of $S_{+}^{\Gamma}(\rho^{\Gamma+}) + S_{-}^{\Gamma}(\rho^{\Gamma-})$ are orthogonal with each other if and only if

$$\langle V_i^{\pm}\tilde{\psi}_j^{\mp} | V_k^{+}\tilde{\psi}_l^{+}\rangle = 0\, \forall i,j,k,l \text{ and} \tag{118}$$

$$\langle V_i^{\pm}\tilde{\psi}_j^{\mp} | V_k^{-}\tilde{\psi}_l^{-}\rangle = 0\, \forall i,j,k,l, \tag{119}$$

the first condition. □

9.2 $2 \otimes 2$ unitary operation

The unitary operator is

$$\begin{pmatrix} \cos\alpha & \sin\alpha & 0 & 0 \\ -\sin\alpha & \cos\alpha & 0 & 0 \\ 0 & 0 & \cos\beta & \sin\beta \\ 0 & 0 & -\sin\beta & \cos\beta \end{pmatrix}.$$

The eigenvalues of $\mathscr{T}(S^{\Gamma})$ are:

$$\{c_i^{+}\} = \{2 \pm 2\cos(\beta - \alpha), 2|\sin(\beta - \alpha)|\},\ c^{-} = -2|\sin(\beta - \alpha)|.$$

For pure and separable state $|\psi\rangle = |\psi_1\rangle|\psi_2\rangle$, (116) becomes

$$(\langle\psi_1^*|\langle\psi_2|)V_i^{-\dagger}V_j^{+}(|\psi_1^*\rangle|\psi_2\rangle) = 0\ \forall i,j. \tag{120}$$

With $|\psi_i\rangle = \cos\theta_i|\uparrow\rangle + e^{i\phi_i}\sin\theta_i|\downarrow\rangle$, depending on the sign of $\sin(\beta - \alpha)$,

$$\{\langle\psi_1^*|\langle\psi_2|V^{-\dagger}V_i^{+}|\psi_1^*\rangle|\psi_2\rangle\} = \{\mp 2i\sec(\beta+\alpha)\cos(2\theta_1)\sin(2\theta_2)\sin\phi_2, 2\frac{\cos(2\theta_1) \pm i\sin(2\theta_2)\sin\phi_2}{-1+\cos(\alpha+\beta)\mp\sin(\alpha+\beta)},$$
$$\mp\sec^2\frac{\beta+\alpha}{2}\frac{\cos(2\theta_1)\mp i\sin(2\theta_2)\sin\phi_2}{\pm 1 + \tan((\beta+\alpha)/2)}\},$$

taking the upper ones when $\sin(\beta - \alpha) \geq 0$. For all of them to vanish, $\theta_1 = \pi/4 + n\pi/2$, and $\theta_2 = m\pi/2$ or $\phi_2 = p\pi$, with $m, n, p \in \mathbb{Z}$.

9.3 $2 \otimes 3$ unitary operation

Consider a $2 \otimes 3$ unitary operator

$$\left(\begin{array}{c|ccc} I_3 & & 0_{3\times3} & \\ \hline & \cos\beta & \sin\beta & 0 \\ 0_{3\times3} & -\sin\beta & \cos\beta & 0 \\ & 0 & 0 & 1 \end{array}\right) \cdot \left(\begin{array}{c|cc} I_4 & \multicolumn{2}{c}{0_{4\times4}} \\ \hline 0_{4\times4} & \cos\alpha & \sin\alpha \\ & -\sin\alpha & \cos\alpha \end{array}\right). \tag{121}$$

For the upper and lower bound to be the same with $\widetilde{S^\Gamma}_- = S^\Gamma_-$, we need ${S^\Gamma}^\dagger_-(I) \propto I$, i.e. the maximum and minimum eigenvalues of ${S^\Gamma}^\dagger_-(I) \propto I$ should be identical. From Fig. 1 they seem to be the same at $1/2$ when $\alpha = 2\pi/3$ and $\beta = 0$, which can indeed be verified analytically, so the exact entangling capacity at $\alpha = 2\pi/3$ and $\beta = 0$ can be acquired.

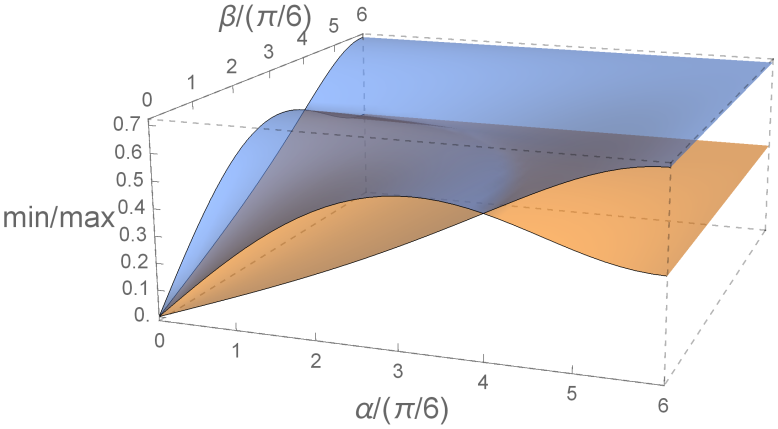

Figure 1: The max/min eigenvalues of ${S^\Gamma_-}^\dagger(I)$, with eigenvalue 0 taken into account.

9.3.1 Degeneracy

When $\alpha = 2\pi/3$ and $\beta = 0$, the spectrum of $\mathscr{T}(S^\Gamma)$ is $\{-3, 3, 3, 3\}$, which has degeneracy. When trying to find the optimal state for it i.e. to solve (120), the eigenvectors with eigenvalue 3 that came out of the program were not orthogonal but were linearly independent, and we did not try to orthogonalize them for the following reason:

The dimension of an eigenspace with a degenerate eigenvalue is higher than one. Suppose the said eigenspace can be spanned by $\{|v_i\rangle\}$ and $\{|\widetilde{v}_i\rangle\}$, both with the same cardinality but the former being an orthonormal set and the latter being normalized but not orthogonal. Hence one is the linear combination of the other:

$$|v_i\rangle = \sum_j c_{ij} |\widetilde{v}_j\rangle, \; c_{ij} \text{ is non-singular.}$$

The Kraus operator corresponding to an eigenvector $|v_i\rangle = \sum_{jk} d^i_{jk} |a_j\rangle |b_k\rangle$ of a linear mapping is

$$V_i := V(|v_i\rangle) := \sum_{j,k} d^i_{jk} |b_k\rangle\langle a_j|, \tag{122}$$

which is a linear process, that is, $V_i = \sum_j c_{ij} \widetilde{V}_j$, where $\widetilde{V}_j := V(|\widetilde{v}_j\rangle)$.

Therefore, $V_i|\psi\rangle = \sum_j c_{ij} \widetilde{V}_j |\psi\rangle$, and $\{V_i|\psi\rangle\}$ has the same span as $\{\widetilde{V}_i|\psi\rangle\}$ does. For two (sub)spaces to be orthogonal, it does not matter which vectors we choose to span them, just as discussed in the proof for Lemma 2. This explains why there is no need to orthogonalize eigenvectors with the same eigenvalue if we just want to find the optimal state.

9.3.2 The solution

Assuming $|\psi\rangle = (\cos\theta_1 |{\uparrow}\rangle + \sin\theta_1 |{\downarrow}\rangle) \otimes (\cos\theta_2 \sin\phi |0\rangle + \sin\theta_2 \sin\phi |1\rangle + \cos\phi |2\rangle)$, for this operation at $\alpha = 2\pi/3$ and $\beta = 0$, (120) becomes

$$0 = 4\sin^2\theta_1 - 3f, \tag{123}$$
$$0 = 4g\sin^2\theta_1 - f, \tag{124}$$
$$0 = \cos^2\theta_1 \left(1 - 3g\right), \tag{125}$$

where

$$f = \cos^2\theta_1 \left(1 + g\right), \; g = \sin^2\phi \cos^2\theta_2.$$

From (125), either $\cos^2\theta_1 = 0$ or $1 - 3g = 0$. However, $\cos^2\theta_1 \neq 0$; otherwise f would vanish, and from (123), $\sin^2\theta_1$ would be zero, a contradiction. Hence, $g = 1/3$, so $f = 4\cos^2\theta_1/3$, and (124) is satisfied. (123) now becomes

$$4\sin^2\theta_1 - 4\cos^2\theta_1 = 0 = -4\cos 2\theta_1.$$

Thus the solution is

$$\sin^2\phi\cos^2\theta_2 = 1/3 \text{ and } \cos 2\theta_1 = 0.$$

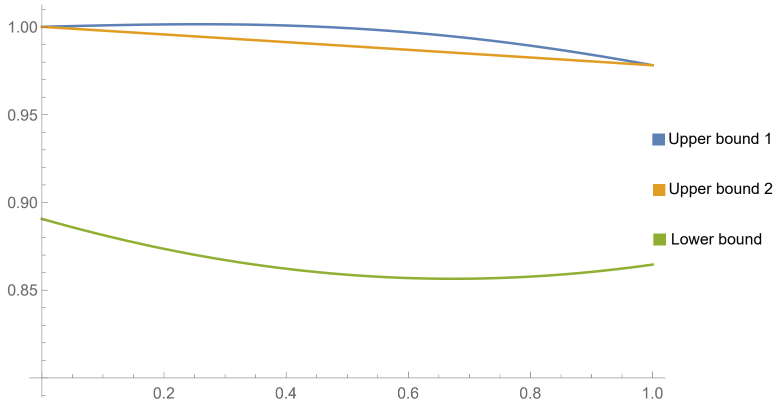

(a) $2 \otimes 3$ operations. Here upper bound 1 is larger than 2 when $0 < p < 1$.

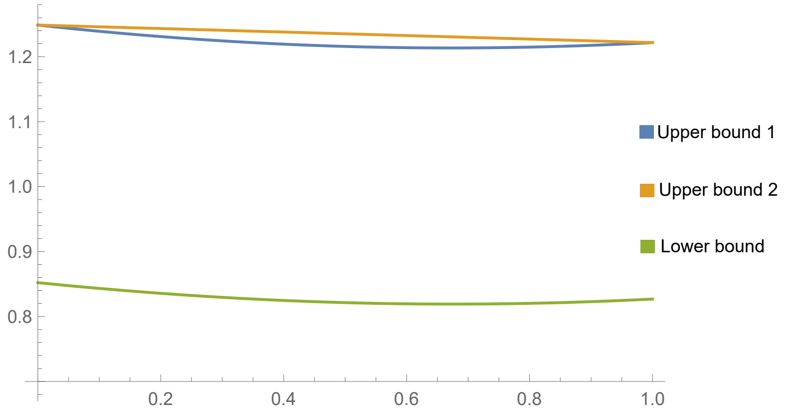

(b) $3 \otimes 3$ operations. Here upper bound 2 is larger than 1 when $0 < p < 1$.

Figure 2: Upper and lower bounds for EC_L (base 2) of operations of the form $S(\rho) = pS_1 + (1-p)S_2$ with unitary S_i, the lower bound accompanied as a reference. For both the $2\otimes 3$ and $3\otimes 3$ operations, S_1 has $\alpha = \pi/3$ and $\beta = \pi/5$, and S_2 has for S_2, $\alpha = \pi/4$ and $\beta = \pi/3$. Upper bound 1 refers to that obtained with S_-^Γ, and upper bound 2 with $pS_{1\,-}^\Gamma + (1-p)S_{2\,-}^\Gamma$. Fig. 2a clearly demonstrate that $S_-^{\Gamma\,\dagger}(I)$ in general doesn't have the smallest operator norm among all $\widetilde{S^\Gamma_-}^\dagger(I)$.

9.4 $S_{i\,-}^\Gamma$ does not necessarily have the smallest operator norm

For a Hermitian operator H, because there exist $\widetilde{H}^\pm$ such that $\widetilde{H}^\pm - H^\pm \not\geq 0$ (see Sec. 2.1.1), $\widetilde{S_{i\,-}^\Gamma} - S_{i\,-}^\Gamma$ is not always CP, and $\widetilde{S_{i\,-}^\Gamma}^\dagger(I) - S_{i\,-}^{\Gamma\,\dagger}(I)$ can be non-positive. Therefore it can happen that $||S_{i\,-}^{\Gamma\,\dagger}(I)|| > ||\widetilde{S_{i\,-}^\Gamma}^\dagger(I)||$; nevertheless, $S_{i\,-}^{\Gamma\,\dagger}(I)$ has the smallest trace (norm), because of Lemma 1 and Corollary 1. How to minimize the operator norm under such decompositions may be an interesting mathematical problem.

To show $||S_{i\,-}^{\Gamma\,\dagger}(I)|| \not\leq ||\widetilde{S_{i\,-}^\Gamma}^\dagger(I)||$ in general, we consider a deterministic operation $S = pS_1 + (1-p)S_2$, where $0 \leq p \leq 1$ and S_i are unitary operations. We choose $2\otimes 3$ and $3\otimes 3$ unitary operations, the former being (121)

and the latter being

$$\left(\begin{array}{c|ccc} I_6 & & 0_{6\times 3} & \\ \hline & \cos\beta & \sin\beta & 0 \\ 0_{3\times 6} & -\sin\beta & \cos\beta & 0 \\ & 0 & 0 & 1 \end{array}\right) \cdot \left(\begin{array}{c|cc} I_7 & & 0_{7\times 2} \\ \hline & \cos\alpha & \sin\alpha \\ 0_{2\times 7} & -\sin\alpha & \cos\alpha \end{array}\right). \tag{126}$$

The result is illustrated by Fig. 2, where we can see $||{S^{\Gamma}}^{\dagger}_{-}(I)|| > ||\widetilde{S^{\Gamma}}^{\dagger}_{-}(I)||$ with $\widetilde{S^{\Gamma}}_{-} = pS^{\Gamma}_{1\,-} + (1-p)S^{\Gamma}_{2\,-}$ in some cases.

References

[1] J.-Y. Kao, arXiv:2008.03893 [quant-ph] (2020), URL https://arxiv.org/abs/2008.03893.

[2] E. Størmer, *Positive linear maps of operator algebras* (Springer, Berlin/Heidelberg, 2013).

[3] B. Kraus and J. I. Cirac, Phys. Rev. A **63**, 062309 (2001), URL https://link.aps.org/doi/10.1103/PhysRevA.63.062309.

[4] M. S. Leifer, L. Henderson, and N. Linden, Phys. Rev. A **67**, 012306 (2003), URL https://link.aps.org/doi/10.1103/PhysRevA.67.012306.

[5] A. Chefles, Phys. Rev. A **72**, 042332 (2005), URL https://link.aps.org/doi/10.1103/PhysRevA.72.042332.

[6] E. T. Campbell, Phys. Rev. A **82**, 042314 (2010), URL https://link.aps.org/doi/10.1103/PhysRevA.82.042314.

[7] L. H. Loomis and S. Sternberg, *Advanced Calculus* (Jones and Bartlett Publishers, Boston, 1990), rev. ed.

[8] L. W. Tu, *An Introduction to Manifolds* (Springer, New York, 2010), 2nd ed.

[9] R. A. Horn and C. R. Johnson, *Matrix Analysis* (Cambridge University Press, Cambridge, 2013), 2nd ed.

[10] A. Jamiołkowski, Rep. Math. Phys. **3**, 275 (1972), ISSN 0034-4877, URL http://www.sciencedirect.com/science/article/pii/0034487772900110.

[11] K. Kraus, Ann. Phys. **64**, 311 (1971), ISSN 0003-4916, URL http://www.sciencedirect.com/science/article/pii/0003491671901084.

[12] M.-D. Choi, Linear Algebra Its Appl. **10**, 285 (1975), ISSN 0024-3795, URL http://www.sciencedirect.com/science/article/pii/0024379575900750.

[13] I. Bengtsson and K. Zyczkowski, *Geometry of Quantum States: An Introduction to Quantum Entanglement* (Cambridge University Press, Cambridge, 2008).

[14] J. de Pillis, Pacific J. Math. **23**, 129 (1967), URL https://projecteuclid.org/euclid.pjm/1102991990.

[15] M. Jiang, S. Luo, and S. Fu, Phys. Rev. A **87**, 022310 (2013), URL https://link.aps.org/doi/10.1103/PhysRevA.87.022310.

[16] E. M. Rains, Phys. Rev. A **60**, 173 (1999), URL https://link.aps.org/doi/10.1103/PhysRevA.60.173.

[17] W. Rudin, *Real and Complex Analysis* (McGraw-Hill, New York, 1987), 3rd ed.

[18] R. Bhatia, *Matrix Analysis* (Springer, New York, 1997).

[19] A. E. Rastegin, J. Stat. Phys. **148**, 1040 (2012), URL https://doi.org/10.1007/s10955-012-0569-8.

[20] B. Baumgartner (2011), math-ph/1106.6189.

[21] G. Vidal and R. F. Werner, Phys. Rev. A **65**, 032314 (2002), URL http://link.aps.org/doi/10.1103/PhysRevA.65.032314.

[22] S. Roman, *Advanced Linear Algebra* (Springer, New York, 2008), 3rd ed.

[23] L. P. Hughston, R. Jozsa, and W. K. Wootters, Phys. Lett. A **183**, 14 (1993), ISSN 0375-9601, URL http://www.sciencedirect.com/science/article/pii/0375960193908809.

[24] P. Horodecki, Phys. Lett. A **232**, 333 (1997), ISSN 0375-9601, URL http://www.sciencedirect.com/science/article/pii/S0375960197004167.

[25] M. B. Plenio, Phys. Rev. Lett. **95**, 090503 (2005), URL http://link.aps.org/doi/10.1103/PhysRevLett.95.090503.

[26] T. M. Cover and J. A. Thomas, *Elements of information theory* (John Wiley & Sons, Inc., Hoboken, 2006), 2nd ed.

[27] D. A. Lidar, P. Zanardi, and K. Khodjasteh, Phys. Rev. A **78**, 012308 (2008), URL https://link.aps.org/doi/10.1103/PhysRevA.78.012308.

[28] M. A. Nielsen, C. M. Dawson, J. L. Dodd, A. Gilchrist, D. Mortimer, T. J. Osborne, M. J. Bremner, A. W. Harrow, and A. Hines, Phys. Rev. A **67**, 052301 (2003), URL https://link.aps.org/doi/10.1103/PhysRevA.67.052301.

[29] K. Życzkowski and I. Bengtsson, Annals of Physics **295**, 115 (2002), ISSN 0003-4916, URL http://www.sciencedirect.com/science/article/pii/S0003491601962013.
